# Complete Reconstitution of the Vancomycin-Intermediate Staphylococcus aureus Phenotype of Strain Mu50 in Vancomycin-Susceptible S. aureus

**DOI:** 10.1128/AAC.00420-16

**Published:** 2016-05-23

**Authors:** Yuki Katayama, Miwa Sekine, Tomomi Hishinuma, Yoshifumi Aiba, Keiichi Hiramatsu

**Affiliations:** a Department of Microbiology, Faculty of Medicine, Juntendo University, Tokyo, Japan; b Research Centre for Infection Control Science, Juntendo University, Tokyo, Japan

## Abstract

Complete reconstitution of the vancomycin-intermediate Staphylococcus aureus (VISA) phenotype of strain Mu50 was achieved by sequentially introducing mutations into six genes of vancomycin-susceptible S. aureus (VSSA) strain N315ΔIP. The six mutated genes were detected in VISA strain Mu50 but not in N315ΔIP. Introduction of the mutation Ser329Leu into *vraS*, encoding the sensor histidine kinase of the *vraSR* two-component regulatory (TCR) system, and another mutation, Glu146Lys, into *msrR*, belonging to the LytR-CpsA-Psr (LCP) family, increased the level of vancomycin resistance to that detected in heterogeneous vancomycin-intermediate S. aureus (hVISA) strain Mu3. Introduction of two more mutations, Asn197Ser into *graR* of the *graSR* TCR system and His481Tyr into *rpoB*, encoding the β subunit of RNA polymerase, converted the hVISA strain into a VISA strain with the same level of vancomycin resistance as Mu50. Surprisingly, however, the constructed quadruple mutant strain ΔIP4 did not have a thickened cell wall, a cardinal feature of the VISA phenotype. Subsequent study showed that cell wall thickening was an inducible phenotype in the mutant strain, whereas it was a constitutive one in Mu50. Finally, introduction of the Ala297Val mutation into *fdh2*, which encodes a putative formate dehydrogenase, or a 67-amino-acid sequence deletion into *sle1* [*sle1*(Δ67aa)], encoding the hydrolase of *N*-acetylmuramyl-l-alanine amidase in the peptidoglycan, converted inducible cell wall thickening into constitutive cell wall thickening. *sle1*(Δ67aa) was found to cause a drastic decrease in autolysis activity. Thus, all six mutated genes required for acquisition of the VISA phenotype were directly or indirectly involved in the regulation of cell physiology. The VISA phenotype seemed to be achieved through multiple genetic events accompanying drastic changes in cell physiology.

## INTRODUCTION

Studies on the genetic mechanism of vancomycin-intermediate resistance revealed that it is acquired by Staphylococcus aureus through multistep mutations of the genes that are involved in the regulation of cell physiology (1–6). When exposed to cell wall synthesis inhibitors, S. aureus upregulates cell wall synthesis through the activation of the *vraSR* two-component regulatory (TCR) system (7–9). We previously reported that not only vancomycin but also β-lactam antibiotics, such as imipenem, can select for heterogeneous vancomycin-intermediate S. aureus (hVISA) mutants from a vancomycin-susceptible S. aureus (VSSA) strain, N315ΔIP (ΔIP) (10). In fact, such selection of hVISA by exposure to β-lactam antibiotics occurred in Japanese hospitals before the clinical introduction of vancomycin (11). Enhanced cell wall synthesis was observed in clinically isolated hVISA strain Mu3 as well as in hVISA strain ΔIP1 (previously designated strain ΔIP-H14 [9]) obtained *in vitro* by selection with imipenem at 8 μg/ml (10). Both strains had mutations in the *vraS* gene, encoding the sensor histidine kinase, which brought about the constitutive expression of the response regulator *vraR* and more than 50 genes which are under the control of the TCR system. The transcription of many genes involved in cell wall synthesis was found to be significantly augmented (9, 10). Therefore, the upregulation of cell wall synthesis caused by activation of the *vraSR* TCR system definitely contributes to vancomycin resistance. Clinical hVISA strain Mu3 and laboratory strain ΔIP1 carry different *vraS* mutations: the Ile5Asn (I5N) mutation in *vraS* [*vraS*(I5N)] and the Ser329Leu (S329L) mutation in *vraS* [*vraS*(S329L)]. However, the mutations had the same effect, i.e., constitutive activation of *vraSR* and upregulation of the genes involved in cell wall synthesis (10).

Such *vraS* mutations are frequently observed in hVISA strains in Japan (12) and may represent first-step mutations leading to the acquisition of the VISA phenotype (10, 13, 14). In a search for the next genetic events leading to the VISA phenotype, we determined and compared the whole-genome sequences of hVISA strain Mu3 and VISA strain Mu50 (15, 16). Nine Mu50-specific nonsynonymous mutations were identified, and among these we found regulator mutation Asn197Ser (N197S) in *graR* [*graR*(N197S)], encoding TCR and the response to host defense peptide systems (16, 17). Introduction and overexpression of the mutated *graR* gene in Mu3 increased the level of vancomycin resistance to that in VISA strains (16). However, the level of vancomycin resistance of Mu3 established by introduction of a single copy of *graR*(N197S) by gene replacement increased the vancomycin MIC, but the strain did not attain the level of resistance defined for vancomycin-intermediate resistance (MIC ≥ 4 μg/ml) (18). We then examined another Mu50-specific mutation, His481Tyr (H481Y) in *rpoB* [*rpoB*(H481Y)], encoding the β subunit of RNA polymerase. We found that introduction of the mutated *rpoB* together with *graR*(N197S) into Mu3 increased its vancomycin MIC to 8 μg/ml, equivalent to that for Mu50 (18). We have already suggested that the *rpoB* mutation is one of the regulatory mutations increasing the level of resistance to vancomycin, daptomycin, and β-lactams (6, 18–22).

Here, we planned to reconstitute the entire VISA phenotype in a naive vancomycin-susceptible methicillin-resistant S. aureus (MRSA) strain which had not been exposed to vancomycin. For this project, we chose laboratory strain N315ΔIP (ΔIP), a laboratory derivative of clinical pre-MRSA strain N315 in which *mecI* was inactivated and the plasmid carrying the gene for penicillinase (PCase; β-lactamase) was eliminated. N315 represents the dominant health care-associated (HA) MRSA strain that has the same sequence type (ST5) as strains Mu3 and Mu50. N315 was isolated in 1982 prior to the clinical introduction of vancomycin in 1991 (11). ΔIP was constructed to mimic Mu3 and Mu50, which have no PCase plasmid and in which the *mecI* genes are inactivated by mutation (23). We constructed a triple mutant strain of ΔIP1 by introducing the three mutations *graR*(N197S), *rpoB*(H481Y), and *vraS*(S329L). Contrary to our expectation, however, this strain did not the express VISA phenotype.

The Glu146Lys (E146K) mutation in *msr* [*msr*(E146K)] was previously shown to lower the imipenem MIC and teicoplanin resistance when it was overexpressed in VSSA strain N315 (3). The *msrR* gene is present on the S. aureus chromosome as one of the three paralogs encoding proteins of the LytR-CpsA-Psr (LCP) family (24). *msrR* (or *lcpA*) and the other two *lcp* genes, *lcpB* and *lcpC*, are proposed to function in the last stage of wall teichoic acid (WTA) synthesis, namely, in the attachment of teichoic acid to peptidoglycan (PG) (24, 25). WTA is proposed to control the autolysis of S. aureus cells through the stabilization of autolysin (26). However, it is not yet clear how the altered MsrR in Mu3 and Mu50 contributes to the rise in vancomycin resistance.

We have already reported that the Ala297Val (A297V) mutation in *fdh2* [*fdh2*(A297V)], encoding a putative mutated formate dehydrogenase, is responsible for resistance to vancomycin (6). The deletion of a 67-amino-acid sequence (Δ67aa) from the *lysM* domain in the *sle1* gene [*sle1*(Δ67aa)], encoding the hydrolase of *N*-acetylmuramyl-l-alanine amidase in the peptidoglycan, was also found in Mu50 (3). It has been reported that the localization of Sle1 to the cross wall is abolished in staphylococcal *tagO* mutants, which are defective for WTA synthesis (27, 28). The teichoic acids regulate peptidoglycan cross-linking through the control of PBP 4 activity (29). We previously reported that the *tagO*, *cmk*, or *rpoB* mutation was found in a VISA strain obtained from an hVISA strain (6, 18). It was suggested that the altered teichoic acid synthesis, reduced peptidoglycan cross-linking, and upregulated cell wall synthesis by the UTP pool are closely associated with the VISA phenotype (1, 30, 31). We identified two novel mutations, *msrR*(E146K) and *sle1*(Δ64aa), associated with WTA synthesis that were required for the complete reconstitution of the VISA phenotype of Mu50.

## MATERIALS AND METHODS

### Bacterial strains, plasmids, growth conditions, and determination of doubling time.

The Staphylococcus strains and plasmids used in the present study are listed in Table 1. The cloning and transformation of Escherichia coli DH5α were carried out by standard techniques (TaKaRa-Bio Co., Ltd., Shiga, Japan). All S. aureus strains were cultivated in brain heart infusion (BHI) broth or agar (Difco Laboratories, Detroit, MI) with aeration at 37°C, unless indicated otherwise. The antibiotics tetracycline and chloramphenicol (Sigma Chemical Co., St. Louis, MO) were used for the selection of the S. aureus transformants. The doubling time was calculated as described previously (19). The growth conditions were 37°C with shaking at 25 rpm in a TN-2612 incubator (Advantec, Tokyo, Japan). The optical density at 660 nm (OD_660_) versus time was plotted for each strain in the exponential growth phase.

**TABLE 1:** Bacterial strains used in this study^*a*^

Strain	Description and relevant phenotype	Reference or source
Clinically isolated strains		
Mu50	VISA clinical strain with *vraS*(I5N), *msrR*(E146K), *graR*(N197S), *rpoB*(H481Y), and *fdh2*(A297V) mutations	13
Mu3	hVISA clinical strain with *vraS*(I5N) and *msrR*(E146K) mutations	13
Control strains		
N315	Pre-MRSA clinical strain carrying a functional *mecI* gene encoding the *mecA* gene transcription repressor; hetero-Met^r^	22
ΔIP	N315ΔIP, N315-derived laboratory strain, *mecI*::*tetL*, cured of PCase plasmid; hetero-Met^r^ Tet^r^	22
ΔIP1	ΔIP-derived strain with *vraS*(S329L) mutation formerly designated ΔIP-H14; hVISA homo-Met^r^ Tet^r^	9, 10
Δ*msrR* derivatives		
ΔIP Δ*msrR*	*msrR*-null mutant obtained from ΔIP; homo-Met^r^ Tet^r^	This study
ΔIP1 Δ*msrR*	*msrR*-null mutant obtained from ΔIP1; homo-Met^r^ Tet^r^	This study
ΔIP derivatives with wild-type *vraS*		
ΔIP-m	*msrR*(E146K) mutant obtained by gene replacement from ΔIP; Tet^r^	This study
ΔIP-mr	*rpoB*(H481Y) mutant obtained by rifampin selection of ΔIP-m; Rif^r^ Tet^r^	This study
ΔIP-g	*graR* (N197S) mutant obtained by gene replacement from ΔIP; Tet^r^	This study
ΔIP-r	*rpoB*(H481Y) mutant obtained by rifampin selection of ΔIP; Rif^r^ Tet^r^	10
ΔIP-gr	*rpoB*(H481Y) mutant obtained by rifampin selection of ΔIP-g; Rif^r^ Tet^r^	This study
ΔIP1 derivatives carrying *vraS*(S329L)		
ΔIP1-g	*graR*(N197S) mutant obtained by gene replacement from ΔIP1; homo-Met^r^ Tet^r^	This study
ΔIP1*-*r	*rpoB*(H481Y) mutant obtained by rifampin selection of ΔIP1; homo-Met^r^ Tet^r^	10
ΔIP1-gr	*rpoB*(H481Y) mutant obtained by rifampin selection of ΔIP1-g; homo-Met^r^ Rif^r^ Tet^r^	This study
ΔIP2	*msrR*(E146K) mutant obtained by gene replacement from ΔIP1; homo-Met^r^ Tet^r^	This study
ΔIP2 derivatives carrying both *vraS*(S329L) and *msrR*(E146K)		
ΔIP2-r	*rpoB*(H481Y) mutant obtained by rifampin selection of ΔIP2; homo-Met^r^ Rif^r^ Tet^r^	This study
ΔIP3	*graR*(N197S) mutant obtained by gene replacement from ΔIP2; homo-Met^r^ Tet^r^	This study
ΔIP4	*rpoB*(H481Y) mutant obtained by rifampin selection of ΔIP3; homo-Met^r^ Rif^r^ Tet^r^	This study
ΔIP4-s	*sle1*(Δ67aa) mutant obtained by rifampin selection of ΔIP4; homo-Met^r^ Rif^r^ Tet^r^	This study
ΔIP5	*fdh2*(A297V)^*b*^ mutant obtained by gene replacement from ΔIP4; homo-Met^r^ Rif^r^ Tet^r^	This study
ΔIP6	*sle1*(Δ67aa) mutant obtained by gene replacement from ΔIP5; homo-Met^r^ Rif^r^ Tet^r^	This study

a Abbreviations: m, *msrR*(E146K); g, *graR*(N197S); r, *rpoB*(H481Y); s *sle1*(Δ67aa); Met^s^, methicillin susceptible; Met^r^, methicillin resistant; Tet^r^, tetracycline resistant; Rif^r^, rifampin resistant.

b *fdh2*, encoding a putative formyl dehydrogenase, corresponds to the open reading frames SA2102 of N315 and SAV2039 of Mu50.

### Antibiotic susceptibility tests.

Antibiotic susceptibility was examined by Etest (AB Biodisk, Solna, Sweden) and population analysis as described previously (32). All examinations were performed by using BHI broth or agar.

### DNA methods.

DNA manipulations were performed by standard methods (33). Restriction enzymes were used as recommended by the manufacturer (TaKaRa). Routine PCR amplification was performed with an Expand high-fidelity system (Roche, Mannheim, Germany).

### Construction of ΔIP and ΔIP1 mutants carrying *msrR*(E146K), *graR* (N197S), *fdh2*(A297V), and *sle1*(Δ67aa).

All mutants tested in this study are described in Tables 1 and 2. For replacement of the asparagine amino acid residue at position 197 in *graR* with serine, replacement of the glutamate at position 146 in *msrR* with lysine, and replacement of the alanine at position 297 in *fdh* (*sa2102*) with valine, we used the pKOR1 allele replacement system, as described previously (10, 34). In brief, 1.0 kb of *graR*, *msrR*, or *sle1* insert DNA encompassing 1-kb flanking sequences of the phage attachment sites was generated by PCR from the chromosomal DNA of strain Mu50 (Table 3). The resulting plasmids, pKO-*graR*(N197S) pKO-*msrR*(E146K), and pKO-Δ*sle1*, were introduced into S. aureus by electroporation. pkO-*fdh*(A297V) has been reported by Matsuo et al. (6). Replacement of the *graR*(N197S) and *msrR*(E146K) gene alleles with wild-type *graR* and *msrR*, respectively, in ΔIP or ΔIP1 was carried out by a two-step procedure, as described previously (10).

**TABLE 2:** Genotypes and antibiograms of ΔIP and ΔIP-derived mutant strains compared with those of Mu3 and Mu50

Strain^*a*^	Genotype^*b*^						MIC (μg/ml) of^*c*^:							VAN phenotype^*d*^
	*vraS*	*msrR*	*graR*	*rpoB*	*fdh2*	*sle1*	VAN	TEC	RIF	OXA	IPM	DAP	LZD	
Clinically isolated strains														
Mu50	I5N	E146K	N197S	H481Y	A297V	Δ67aa^*e*^	12	12	>32	>256	>32	3	0.5	VISA
Mu3	I5N	E146K	—	—	—	—	3	24	0.006	>256	>32	2	0.75	hVISA
Control strains														
ΔIP	—	—	—	—	—	—	1	1	0.004	6	0.75	0.75	1	VSSA
ΔIP1	S329L	—	—	—	—	—	2	8	0.004	>256	>32	2	0.75	hVISA
Δ*msrR* derivatives														
ΔIP Δ*msrR*	—	Deletion	—	—	—	—	1	0.75	0.004	2	0.19	1	0.75	VSSA
ΔIP1 Δ*msrR*	S329L	Deletion	—	—	—	—	2	8	0.004	64	>32	2	0.5	hVISA
ΔIP derivatives with wild-type *vraS*														
ΔIP-g	—	—	N197S	—	—	—	1	0.75	0.006	6	0.38	1.5	1	VSSA
ΔIP-r	—	—	—	H481Y	—	—	1	1	>32	5	1	0.5	0.5	VSSA
ΔIP-gr	—	—	N197S	H481Y	—	—	1.5	1.5	>32	2	0.38	1.5	0.5	VSSA
ΔIP-m	—	E146K	—	—	—	—	1	1	0.004	5	0.5	0.19	1	VSSA
ΔIP-mr	—	E146K	—	H481Y	—	—	1	1	>32	3	0.5	0.5	0.75	VSSA
ΔIP1 derivatives carrying *vraS*(S329L)														
ΔIP1-g	S329L	—	N197S	—	—	—	3	6	0.006	>256	>32	2.5	0.75	hVISA
ΔIP1-r	S329L	—	—	H481Y	—	—	3	12	>32	>256	>32	2	0.38	hVISA
ΔIP1-gr	S329L	—	N197S	H481Y	—	—	3	6	>32	256	32	1.5	0.38	hVISA
ΔIP2	S329L	E146K	—	—	—	—	3	12	0.004	>256	>32	2	0.5	hVISA
ΔIP2 derivatives carrying both *vraS*(S329L) and *msrR*(E146K)														
ΔIP2-r	S329L	E146K	—	H481Y	—	—	6	18	>32	>256	>32	2	0.38	VISA
ΔIP3	S329L	E146K	N197S	—	—	—	4	12	0.003	192	>32	2	0.5	VISA
ΔIP4	S329L	E146K	N197S	H481Y	—	—	12	12	>32	>256	>32	4	0.38	VISA
ΔIP4-s	S329L	E146K	N197S	H481Y	—	Δ67aa^*e*^	12	16	>32	>256	>32	4	0.38	VISA
ΔIP5	S329L	E146K	N197S	H481Y	A297V	—	12	16	>32	256	>32	4	0.38	VISA
ΔIP6	S329L	E146K	N197S	H481Y	A297V	Δ67aa	12	12	>32	>256	>32	4	0.38	VISA

a Abbreviations: m, *msrR*(E146K); g, *graR*(N197S); r, *rpoB*(H481Y); s *sle1*(Δ67aa).

b The position of the amino acid substitution in each gene is indicated. —, wild type (no mutation).

c Abbreviations: VAN, vancomycin; TEC, teicoplanin; RIF, rifampin; OXA, oxacillin; IPM, imipenem; DAP, daptomycin; LZD, linezolid.

d VAN phenotype, category of vancomycin susceptibility: VSSA, vancomycin MIC of <2 mg/liter; VISA, vancomycin MIC of 4 or 8 mg/liter; hVISA, vancomycin susceptibility judged by the shape of the population curve (Fig. 1).

e A sequence of 67 amino acids of Sle1 was deleted from Mu50.

**TABLE 3:** Synthetic oligonucleotide primers

Plasmid constructed and primer designation	Sequence^*a*^ (5′-3′)	Gene^*b*^
pKOR1-*graR*(N197S)		
attB1-*graR*-FW	GGGGACAAGTTTGTACAAAAAAGCAGGCTGTATTGAAGATTTCGGCAAAGTAATGGATACA	*sav0659*
attB2-*graR*-RV	GGGGACCACTTTGTACAAGAAAGCTGGGTATAATCAACTGTATGACGTT	*sav0659*
pKOR1-*msrR*(E146K)		
attB1-*msrR*-FW	GGGGACAAGTTTGTACAAAAAAGCAGGCTACAAGGTGGACAATCAAGAACAGATTCTATCATGGTTGTTC	*sav1362*
attB2-*msrR*-RV	GGGGACCACTTTGTACAAGAAAGCTGGGTTCGTCAATGTAAAATTATTAGAAGGTCGTTCGGATGAACAAT	*sav1362*
pKOR1-Δ*sle1*		
attB1-Δ*sle1*-FW	GGGGACAAGTTTGTACAAAAAAGCAGGCTAGTAATTGCAGCTATTATTGGGACAAGCGCGATTAGCGCTGTTGCGGCA	*sav0465*
attB2-Δ*sle1*-RV	GGGGACCACTTTGTACAAGAAAGCTGGGTAGTTTAGGATTCAATCCAACTTTTCAGCTTGTGAAATGTA	*sav0465*

a Underlining indicates *att* sequences introduced into the primer.

b The genes listed are specific for the chromosome of strain Mu50 (GenBank accession no. AP003367).

### Growth curve and doubling time.

A total of 10^5^ CFU of the preculture was inoculated into 10 ml of BHI broth and incubated at 37°C with shaking at 25 rpm in a model TN-2612 incubator (Advantec). The doubling time was calculated from the slope of the line obtained from a semilogarithmic graph of the growth curve. The log_2_ OD_660_ versus time was plotted for each strain in the exponential growth phase. The doubling time was calculated by the following formula: [(*t*_2_ − *t*_1_) × log_2_]/(log_2_ OD_660_ at *t*_2_ − log_2_ OD_660_ at *t*_1_), where *t*_2_ and *t*_1_ are the times at the end and the start of the logarithmic growth phase, respectively. The doubling time was measured in at least three independent experiments.

### Isolation of a rifampin-resistant mutant strain carrying *rpoB*(H481Y).

Twenty rifampin-resistant isolates were obtained from each of seven strains, strains ΔIP, ΔIP-m, ΔIP-g, ΔIP1, ΔIP1-g, ΔIP2, and ΔIP3, by selection with BHI agar containing 1 μg/ml of rifampin at frequencies of 1.4 × 10^−7^, 3.0 × 10^−8^, 4.0 × 10^−8^, 8.0 × 10^−7^, 6.4 × 10^−8^, 6.5 × 10^−8^, and 9.4 × 10^−8^, respectively. The nucleotide sequences of the *rpoB* genes from the resulting 20 rifampin-resistant isolates were determined by sequencing analysis.

### Transmission electron microscopy.

The preparation of S. aureus cells for transmission electron microscopy and examination of the cells by transmission electron microscopy were performed as described previously (1, 17). At least 100 cells cut nearly equatorially were measured for the evaluation of cell wall thickness, and the results are expressed as the means ± standard deviations.

### Autolysis assay.

Triton X-100-stimulated autolysin activity in Tris-HCl buffer (pH 7.5) was measured as described previously (35). Cells were grown to the mid-exponential phase to an OD_660_ of about 1.5 at a cultivation temperature of 37°C. The culture was rapidly chilled, and the cells were washed twice with ice-cold distilled water and suspended to an OD_660_ of 2.0 in 50 mM Tris-HCl buffer supplemented with 0.05% Triton X-100. Autolytic activity was measured during incubation at 30°C as a decrease in the OD_660_ by using a biophotorecorder (Advantec). All data from the autolysis experiments are reported as percentages of the initial turbidity (at zero time) and are representative of those from three independent experiments.

### RNA preparation and microarray analysis.

RNA extraction, cDNA labeling, hybridization, and volcano plot analysis performed by microarray analysis were carried out according to protocols described previously (19). Using the three normalized signal intensities, the statistical significance of the data was evaluated by Student's *t* test. Ratios of the fold change were calculated by using the average of the normalized signal intensity. Volcano plot analysis was performed as described previously (19), and plots were constructed by plotting the negative log_10_ of the *P* value obtained by the *t* test on the *y* axis and the log_2_ value of the fold change on the *x* axis.

### Statistical analysis.

The two-by-two contingency tables were evaluated by Fisher's exact test.

### Microarray data accession numbers.

The transcriptional profiles were published in NCBI under GEO accession number GSE43643.

## RESULTS

### Properties of ΔIP-derived strains into which *vraS*, *graR*, and *rpoB* mutations are sequentially introduced.

To recreate the VISA phenotype of Mu50, ΔIP-derived strains were constructed by sequentially replacing the *vraS*, *graR*, and *rpoB* genes of ΔIP by their mutated counterparts from Mu50. The *vraS* and *graR* genes of ΔIP were replaced with mutated *vraS* and *graR* by an allele replacement procedure, and *rpoB*(H481Y) was incorporated by rifampin selection. The antibiograms of the strains were determined by Etest (Table 2). We have already reported that strain ΔIP1 has increased vancomycin resistance, and a typical population curve for the hVISA strain is shown in Fig. 1 (10). The replacement of the *graR* and *rpoB* genes in clinical hVISA strain Mu3 with *graR*(N197S) and *rpoB*(H481Y) converted Mu3 into a VISA strain comparable to Mu50 (16, 17). Therefore, we expected that consecutive incorporation of three mutations, *vraS*(S329L), *graR*(N197S), and *rpoB*(H481Y), would convert ΔIP into a VISA strain. Unfortunately, the vancomycin MICs for the resultant ΔIP1 derivatives carrying *vraS*(S329L), *graR*(N197S), and *rpoB*(H481Y) (ΔIP1-gr, ΔIP1-g, and ΔIP1-r, respectively) were not the same as the vancomycin MIC of Mu50 (Table 2).

**FIG 1:**
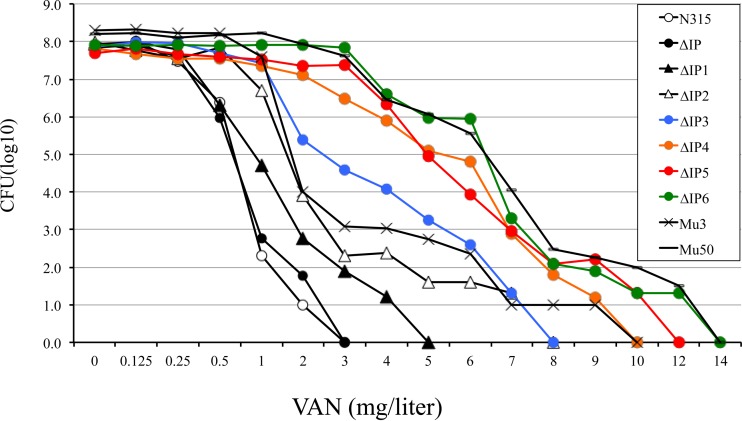
Population analysis of the susceptibility profiles of the vancomycin (VAN)-resistant subpopulation. The profiles of ΔIP, its derivatives, Mu3, and Mu50 were evaluated after 72 h of incubation at 37°C. The sequential introduction of six mutations, resulting in strains ΔIP1, ΔIP2, ΔIP3, ΔIP4, ΔIP5, and ΔIP6, reconstituted the VISA phenotype of Mu50 in VSSA strain N315ΔIP. About 10^7^ CFU of the overnight culture of each strain was inoculated on BHI agar plates containing various concentrations of vancomycin.

### Introduction of *msrR*(E146K) into ΔIP1 converted it to the VISA phenotype with a vancomycin MIC of 4 μg/ml.

We recollected ourselves and looked for other candidate single nucleotide polymorphisms (SNPs) between VSSA strain ΔIP and hVISA strain Mu3. We inferred the presence of another mutation contributing to the increase in vancomycin resistance since there was a substantial difference in the vancomycin MICs for Mu3 (MIC = 3 μg/ml) and ΔIP (MIC = 1 μg/ml) (Table 2). The criteria for the candidate mutations were that they had to be (i) absent from the N315 genome (which has the same chromosome as ΔIP except for a *mecI* deletion) (23) and (ii) shared by both Mu3 and Mu50. However, there were at least 174 SNPs between N315 and Mu50 (15, 19). Therefore, we first looked for the genes in Mu50 which we have identified to be capable of increasing the level of vancomycin resistance when expressed in excess in N315 (30). Among the 17 genes identified, we noticed that *msrR*(E146K) was present in Mu3 and Mu50 but that wild-type *msrR* was present in N315.

**FIG 2:**
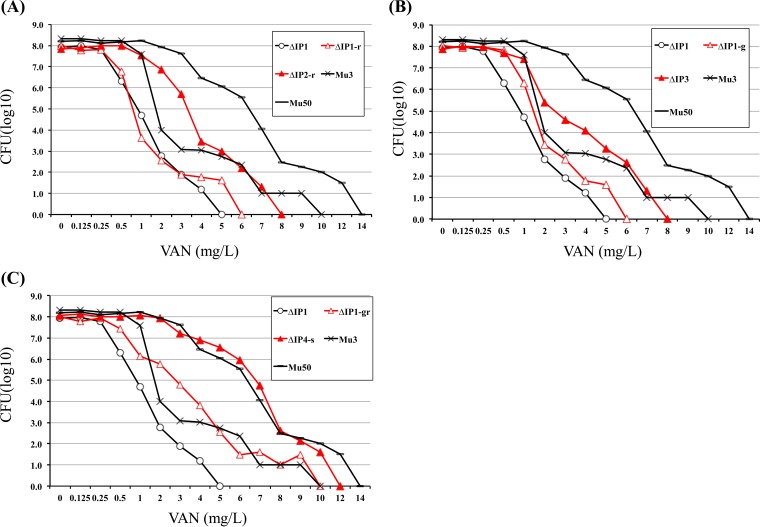
The *msrR*(E146K) mutation confers increasing resistance to vancomycin. The profiles of the resistant subpopulations of strain ΔIP, its derivative mutants, hVISA Mu3, and VISA Mu50 are shown. The number of cells (in log_10_ CFU per milliliter) growing on BHI agar containing vancomycin are shown on the *y* axis. Vancomycin concentrations are shown on the *x* axis. The number of the colonies that grew was counted after incubation at 37°C for 48 h. Red open triangles and red closed triangles, the strain with wild-type *msrR* and the *msrR*(E146K) mutant, respectively.

First, the increase in the vancomycin MIC was not seen in the ΔIP-derived mutants carrying wild-type *vraS* (Table 2). Therefore, the *vraS* mutation was considered an essential genetic event leading to acquisition of the VISA phenotype of Mu50, and without which mutation of the *msrR*, *graR*, or *rpoB* gene or combinations of these genes was not effective in raising the level of vancomycin resistance equivalent to that in Mu50 (Table 2). The first-step mutation *vraS*(I5N) or *vraS*(S329L) converted ΔIP (vancomycin MIC = 1 μg/ml) into pre-hVISA strain ΔIP1 for which the vancomycin MIC was 2 μg/ml. When *msrR*(E146K) was introduced as the second mutation, the phenotype of strain ΔIP2 was converted to the hVISA phenotype and the vancomycin MIC was increased to 3 μg/ml (Table 2). The shape of the curve for the ΔIP2 population was equivalent to that for Mu3 (Fig. 1). Second, when the population analysis for vancomycin susceptibility between the *msrR*(E146K) mutant and its parent strain with wild-type *msrR* were compared, the three *msrR*(E146K) mutants ΔIP2-r, ΔIP3, and ΔIP4 shifted to the right (Fig. 2A to C). In contrast, the oxacillin MICs for Δ*msrR* mutants ΔIP Δ*msrR* and ΔIP2 decreased (Table 2). Thus, these findings indicate that *msrR*(E146K) and *vraS*(S329L) led to increased resistance to vancomycin to a level similar to that in Mu3.

The vancomycin MIC for quadruple mutant ΔIP4 was 12 μg/ml, which corresponded to the MIC for VISA strain Mu50 (Table 2). ΔIP4 also exhibited a population curve similar to that of Mu50 (Fig. 1). Therefore, the introduction of four mutated genes, *vraS*, *msrR*, *graR*, and *rpoB*, into a VSSA strain reconstituted the VISA phenotype.

### Cell wall thickening of ΔIP4 is an inducible phenotype.

We consider cell wall thickening to be a cardinal feature of the VISA phenotype that is directly associated with the clogging mechanism of vancomycin resistance (2, 36). As shown in Table 4, the *vraS*(S329L) mutation thickened the cell wall of ΔIP so that the thickness of its cell was comparable to that of the cell wall of clinical hVISA strain Mu3. ΔIP2 carrying both the *msrR*(E146K) and *vraS*(S329L) mutations showed a thickened cell wall (34.7 nm); however, VISA strain Mu50 had a much thicker cell wall (38.5 nm) (Table 4).

**TABLE 4:** Prolonged doubling time, thickening of cell wall, and autolytic activity in ΔIP derivative strains

Strain^*a*^	Phenotype	Doubling time (min)	Autolysis (% decrease of OD)^*c*^	Cell wall thickness (nm)	
				Without vancomycin	With VAN10^*e*^
Clinical isolates and control strains					
ΔIP	VSSA	26.2	68.1	19.4 ± 2.9	18.5 ± 1.3
ΔIP1	hVISA	28.3	51.1	30.8 ± 4.3	23.7 ± 2.1
Mu3	hVISA	35.7^*b*^	55.5	28.7 ± 3.2	25.7 ± 1.8
Mu50	VISA	37.1^*b*^	26.9	38.5 ± 4.8^*d*^	36.0 ± 1.8^*d*^
ΔIP-derived mutants					
ΔIP-m	VSSA	29.8	NT^*g*^	17.5 ± 1.0	NT
ΔIP-g	VSSA	30.5	NT	NT	NT
ΔIP-r	VSSA	29.7	NT	NT	NT
ΔIP-gr	VSSA	30.3	NT	NT	NT
ΔIP-mr	VSSA	30.6	NT	NT	NT
ΔIP1-derived mutants					
ΔIP1-g	hVISA	28.0	NT	26.7 ± 2.4	NT
ΔIP1-r	hVISA	29.5	NT	25.1 ± 1.6	NT
ΔIP2	hVISA	27.6	21.2	34.7 ± 4.4^*d*^	30.8 ± 3.6
ΔIP1 Δ*msrR*	VSSA	31.4	36.1	Unmeasurable^*f*^	Unmeasurable
ΔIP2-derived mutants					
ΔIP3	VISA	27.8	57.3	32.8 ± 3.8	28.7 ± 4.1
ΔIP4	VISA	37.4^*b*^	0.15	31.6 ± 3.3	35.8 ± 2.0^*d*^
ΔIP4-s	VISA	35.0	0.3	40.9 ± 3.6^*d*^	41.5 ± 2.9^*d*^
ΔIP5	VISA	36.3^*b*^	0.7	36.7 ± 3.2^*d*^	33.0 ± 2.8^*d*^
ΔIP6	VISA	37.6^*b*^	27.9	39.7 ± 4.5^*d*^	46.1 ± 4.8^*d*^

a Abbreviations: m, *msrR*(E146K); g, *graR*(N197S); r, *rpoB*(H481Y); s, *sle1*(Δ67aa).

b *P* < 0.001 for the difference in doubling time from that for ΔIP.

c Triton X-100-induced autolysis of the representative strain. The data represent the percent decrease in the OD_660_ after 4 h of incubation.

d *P* < 0.001 for the difference in length from that for ΔIP1 (previously designated strain ΔIP-H14).

e The thickness of the cell wall was measured after induction with vancomycin at 10 μg/ml (VAN10) for 10 min.

f The photograph taken by transmission electron microscopy is shown in Fig. 6.

g NT, not tested.

**FIG 3:**
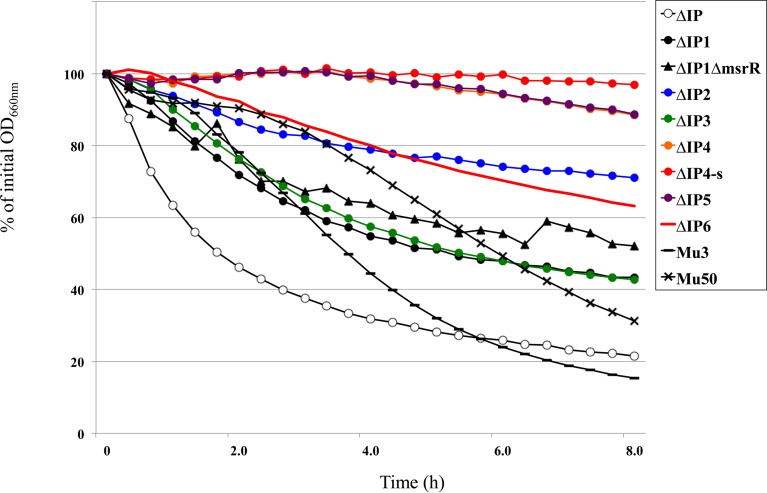
Reduction of autolytic activity in ΔIP mutant strains. The Triton X-100-stimulated autolysis of the parental strain (ΔIP), its derivatives, hVISA Mu3, and Mu50 is shown. Cultures were suspended in autolysis buffer to an initial OD_660_ of 2.0, and the rates of autolysis were monitored as the decrease in OD_660_ with time. The activities of strains ΔIP2, ΔIP4, ΔIP4-s, and ΔIP5 were decreased compared with those of the parent strains, other mutants, hVISA Mu3, and VISA Mu50. Autolysis was measured as the decline in the optical density versus time and is expressed as the percentage of the initial optical density. The percent decrease in OD_660_ after 4 h of incubation is shown in Table 4.

We expected our ΔIP4 construct to have a cell wall as thick as that of Mu50. Contrary to our expectation, however, the thickness of the cell wall of ΔIP4 was only 31.6 nm, which was almost the same as that of the cell wall of ΔIP1 (30.8 nm) and hVISA strain Mu3 (28.7 nm) (Table 4). To reconcile the contradictory observations of vancomycin resistance and a thin cell wall, we considered the possibility of the induction of thickening of the cell wall of ΔIP4. The cell wall of ΔIP4 increased from 31.6 to 35.8 nm after 10 min of exposure to 10 μg/ml of vancomycin. This cell wall thickness corresponded to the cell wall thickness (38.5 nm) of Mu50 exposed to vancomycin. Curiously, ΔIP4 was the only strain tested whose cell wall increased in thickness. The thickness of the cell walls of the other strains, including Mu50, decreased in response to vancomycin. However, clinical VISA strains, including Mu50, have a constitutively thickened cell wall. Therefore, we looked for another mutation in Mu50 that was responsible for the constitutive increase in cell wall thickness. There are nine nonsynonymous SNPs between Mu3 and Mu50 (3). *fdh2*(A297V) (*fdh2* is the ortholog of *sa2102* of N315), encoding a putative mutated formate dehydrogenase (Fdh), was among these SNPs. We designated the mutated gene *fdh2*, since another *fdh* gene presumably encodes Fdh in Mu50 (6). Another candidate SNP is *sle1*, encoding *N*-acetylmuramoyl-l-alanine amidase. A deletion of 67 amino acids (Δ67aa) from the *lysM* domain was found in the *sle1* gene of Mu50 (3). Sle1 attachment to the cross wall is abolished in staphylococcal *tagO* mutants, which are defective for WTA synthesis (27, 28). We introduced the *sle1*(Δ64aa) or *fdh2*(A297V) gene into ΔIP4 or ΔIP5 and then obtained strain ΔIP4-s or ΔIP6 to test the effects of the genes on cell wall thickness, respectively. The vancomycin MIC did not change, nor did the MICs of the other antibiotics (Table 2 and Fig. 1). However, significant cell wall thickening was observed in ΔIP4-s and ΔIP6 (cell wall thicknesses, 40.9 nm and 39.7 nm, respectively) compared to the cell wall thicknesses of their parent strains, ΔIP4 and ΔIP5, respectively (Table 4). The six mutants of ΔIP attained the VISA phenotype of Mu50 with a constitutive increased cell wall thickness (Fig. 3).

### Progressive decrease in growth rate and autolytic activity of the mutant strains which showed increased vancomycin resistance.

A low growth rate is a characteristic feature of strains with the VISA phenotype. Their growth curves showed a shift toward the right as the numbers of mutations increased. The calculated doubling time also increased with the increase in vancomycin resistance of the strains (Table 4). Among the six mutations, *rpoB*(H481Y) had a pronounced effect on slowing the growth of strains ΔIP4, ΔIP5, and ΔIP6 (Table 4).

**FIG 4:**
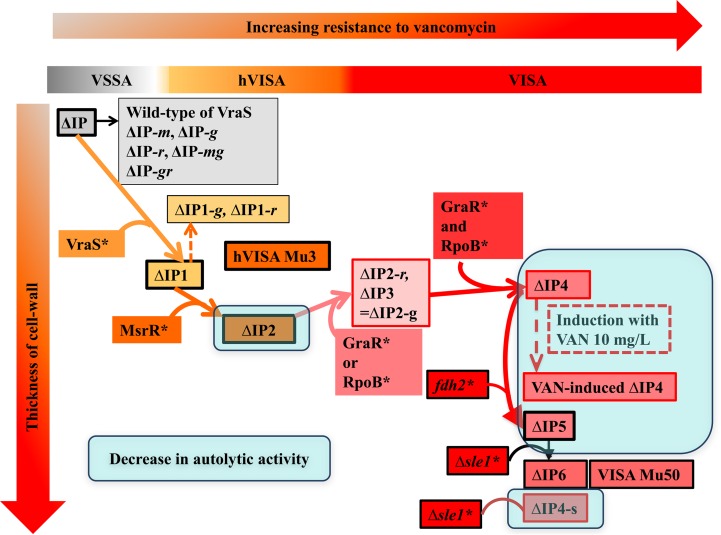
Steps in which phenotypic Mu50-like VISA is reconstituted from VSSA parent strain ΔIP. The increase in resistance to vancomycin was related to the two phenotypes' cell wall thickness and autolytic activity among the ΔIP isogenic laboratory strains. The cell wall thickness is shown on the *y* axis. The level of vancomycin resistance is shown on the *x* axis. The blue areas describe a mutant strain with decreased autolytic activity.

Figure 4 illustrates the results of the assay of autolysis in the presence of 0.05% Triton X-100. We compared the autolytic activity with the percent decrease in the OD_660_ after 4 h of incubation shown in Table 4. A decrease in the rate of cell lysis was observed with the sequential introduction of mutations. When the autolysis activities of *msrR*(E146K) mutant ΔIP2 and the ΔIP1 Δ*msrR* strain were compared, the level of activity in ΔIP2 decreased compared to that in its parent strain, ΔIP1, but it was not changed in the ΔIP1 Δ*msrR* mutant. This finding indicates that *msrR*(E146K) is associated with a decrease in autolytic activity. After 4 h, the ΔIP2, ΔIP4, and ΔIP5 mutants showed a decrease in autolytic activity; however, the autolytic activity in ΔIP6 was similar to that in Mu50 (Table 4).

### Effects of *msrR*(E146K), Δ*msrR*, and *sle1*(Δ64aa) on morphology and cell separation phenotypes.

To investigate the effects of the *msrR*(E146K) and *sle1*(Δ64aa) genes on phenotypic expression, we constructed three mutant strains, ΔIP Δ*msrR*, ΔIP1 Δ*msrR* and ΔIP4-s, and then compared their phenotypes to those of strains ΔIP1, ΔIP2, and ΔIP5. In the absence of vancomycin, overnight cultures of the ΔIP Δ*msrR*, ΔIP1 Δ*msrR*, and ΔIP4-s mutant strains aggregated (see Fig. S1 in the supplemental material). When analyzed by transmission electron microscopy, those cells failed to separate and showed an abnormal morphology. In contrast, the culture and cells of ΔIP2, ΔIP5, and their parent strains, ΔIP and ΔIP4, respectively, showed a normal morphology. Interestingly, ΔIP5, carrying both the *msrR*(E146K) and *sle1*(Δ64aa) mutations, showed increased resistance to vancomycin and reduced autolytic activity and maintained normal separation and a normal morphology. This finding indicates that the *fdh*(A297V) mutation allowed the abnormal separation caused by *sle1*(Δ64aa) in ΔIP5 to be recovered.

### Effects of increasing vancomycin resistance on the relationship between mutated *rpoB* and three mutated genes, *vraS*, *msrR*, and *graR*.

The *rpoB*(H481Y) mutation was also related to the three phenotypes of increasing resistance to vancomycin (Table 2), slow growth (Table 4), and decreased autolytic activity (Fig. 4), as we have reported previously (17). However, the effects of increasing vancomycin resistance on the interaction between *rpoB*(H481Y) and the other three mutated genes, *vraS*(S329L), *msrR*(E146K), and *graR*(N197S), are unknown. To clarify the interaction, we investigated the rate of appearance of hVISA or VISA among the *rpoB*(H481Y) mutants when the ΔIP and ΔIP1 mutants were selected by rifampin. We isolated 20 rifampin-resistant isolates carrying the *rpoB* mutation from each of six mutants, ΔIP-m, ΔIP-g, ΔIP1, ΔIP2, ΔIP1-g, and ΔIP3, and parent strain ΔIP and then determined the MIC of vancomycin by the Etest (Table 5). Interestingly, each of the 20 *rpoB* mutant strains showed a variety of MICs. For 2 (10%), 3 (15%), 20 (100%), 1 (5%), 11 (55%), 3 (15%), and 12 (60%) of 20 of the *rpoB* mutant isolates of ΔIP, ΔIP-m, ΔIP-g, ΔIP1, ΔIP2, ΔIP1-g, and ΔIP3, respectively, the vancomycin MIC was increased compared to that for the parent strain. Among these isolates, 11 (55%) and 3 (15%) of the *rpoB* mutants of hVISA strains ΔIP2 and ΔIP1-g, respectively, changed to VISA (vancomycin MIC ≥ 4 μg/ml). In particular, 3 (15%) *rpoB* mutants of ΔIP3 showed phenotypic expression of Mu50-like VISA resistance (vancomycin MIC = 8 μg/ml). Therefore, it was revealed that a genetic background of all three mutations in *vraS*, *msrR*, and *graR* was needed for conversion of Mu50-like VISA phenotype induced by mutated *rpoB*.

**TABLE 5:** Distribution of amino acid substitutions in *rpoB* among ΔIP mutants by selection with rifampin

Strain^*a*^	Vancomycin MIC (mg/liter)	No. of mutants selected with rifampin with the following mutation in *rpoB*^*b*^:										
		Q468K	Q468L	Q468R	D471V	N474K	A477D	H481R	H481Y	R484H	S486L	Total
ΔIP	1	0	0	1 (1, 0, 0)	0	0	0	0	8 (3, 3, 2)	2 (0, 2, 0)	9 (0, 9, 0)	20 (4, 14, 2)
ΔIP-m	1	0	0	0	0	3 (0, 2, 1)	0	0	2 (0, 1, 1)	2 (0, 1, 1)	13 (1, 12, 0)	20 (1, 16, 3)
ΔIP-g	1	0	0	0	0	0	0	0	5 (0, 0, 5)	0	15 (0, 0, 15)	20 (0, 0, 20)
ΔIP1	2	12 (7, 5, 0)	0	0	0	0	0	0	7 (5, 1, 1)	0	1 (0, 1, 0)	20 (12, 7, 1)
ΔIP2	3	3 (2, 1, 0)	0	0	0	0	0	0	8 (0, 2, 6)	0	9 (0, 4, 5)	20 (2, 7, 11)
ΔIP1-g	3	1 (0, 0, 1)	0	0	1 (0, 1, 0)	0	3 (0, 2, 1)	0	1 (0, 1, 0)	0	14 (3, 10, 1)	20 (3, 14, 3)
ΔIP3	4	0	6 (0, 2, 4)	0	1 (0, 1, 0)	0	1 (0, 0, 1)	1 (0, 0, 1)	6 (0, 1, 5)	0	5 (0, 4, 1)	20 (0, 8, 12)
Total		16 (9, 6, 1)	6 (0, 2, 4)	1 (1, 0, 0)	2 (0, 2, 0)	3 (0, 2, 1)	4 (0, 2, 2)	1 (0, 0, 1)	37 (8, 9, 20)	4 (0, 3, 1)	66 (4, 40, 22)	140 (22, 66, 52)

a Abbreviations: m, *msrR*(E146K); g, *graR*(N197S).

b Selection was with rifampin at 1 μg/ml. Data are for 20 mutants of each strain. The three values in parentheses are the numbers of mutants with vancomycin MICs that were decreased, invariant, or increased, respectively, compared with the vancomycin MIC for the parent strain.

### Transcriptional profiles of genes related to peptidoglycan and WTA synthesis among ΔIP-derived strains and control strains.

The transcriptional profiles of the ΔIP-derived strains were analyzed (GEO accession no. GSE43643). The levels of the transcripts of 51 genes encoding peptidoglycan and WTA synthesis and the 6 genes *vraSR*, *msrR*, *graR*, *rpoB*, *fdh2*, and *sle1* in mutant strain ΔIP1were compared to those in ΔIP (see Fig. S2 and S3 in the supplemental material). Of the 51 genes encoding peptidoglycan and WTA synthesis, 10 genes were upregulated by the presence of the *vraS*(S329L) mutation (see Fig. S2 in the supplemental material). Of these 10 upregulated genes, the *vraSR*, *msrR*(E146K), and *murZ* (encoding UDP-*N*-acetylglucosamine 1-carboxyvinyltransferase) genes were upregulated in the ΔIP2, ΔIP3, and ΔIP4 *msrR*(E146K) mutants compared to their levels of transcription in the strain with wild-type *msrR*. However, the other 9 genes associated with the cell wall synthesis stimulon were downregulated (see Fig. S2A in the supplemental material). The data indicate that the *msrR* mutation allowed the recovery of the level of transcription of several genes that were up- or downregulated by the *vraS* mutation.

In contrast, the transcriptional profiles of the ΔIP5 strain were different from those of the other strain ΔIP-derived strains. The levels of transcription of *fdh2*(A297V), *vraS*(*S329L*), *vraR*, *murF*, *murZ*, *murG*, *murQ*, SA0511, SAA0522, and *capG* genes increased by greater than 2-fold in ΔIP5 compared to their levels of transcription in ΔIP (see Fig. S2A in the supplemental material). The *murZ*, *murQ* (encoding *N*-acetylmuramic acid 6-phosphate esterase), *sa0523* [encoding poly(glycerol-phosphate) α-glucosyltransferase], *sa0524* (encoding GTP cyclohydrolase), *sa0511* (encoding GDP-mannose 4,6-dehydratase), and *capG* (encoding the capsular polysaccharide synthesis protein Cap5G) genes were upregulated in ΔIP5, and upregulation of these genes is common to Mu50 (see Fig. S2A and S3A in the supplemental material). The transcriptional profiles of ΔIP5 were not similar to those of the other ΔIP-derived strains, hVISA strain Mu3, and slow VISA strain ΔIP *rpoB*(R512P), except for the upregulation of the *vraSR* and *murZ* genes (19).

### Transcriptional profiles of 19 genes related to the pyrimidine metabolic pathway.

During the course of the study, the downregulation of numerous members of the pyrimidine operon, comprising *pyrAB*, *pyrB*, *pyrC*, *pyrE*, *pyrF*, *pyrG*, and *pyrH*, as well as that of the regulator *pyrR*, was detected in ΔIP4, ΔIP5, and Mu50 carrying *rpoB*(H481Y) (see Fig. S2B and S3B in the supplemental material).

Moreover, the *cmk* gene, encoding cytidylate kinase, was downregulated in strains ΔIP5 and Mu50. It has been reported that the reduced cytidylate kinase activity in *cmk* mutant strains contributes to the conversion from the hVISA phenotype to the VISA phenotype by thickening the cell wall and reducing the cell growth rate (6). It was indicated that the *pyr* operon and *cmk* were downregulated and the *ndk* gene (encoding nucleoside diphosphate kinase) was upregulated by the presence of *fdh2*(A297V) (see Fig. S2B in the supplemental material).

### Transcriptional profiles of 27 genes related to the pathway from pentose phosphate to cell wall synthesis by way of glycolysis.

To investigate why an increase in the thickness of the cell wall of the ΔIP4 mutant strain was induced by vancomycin, the transcriptional profiles of cells in cultures with and without vancomycin were compared. In the ΔIP1 strain, the *glmS*, *sa0529*, and *sa0528* genes, which are associated with the metabolic pathway from d-ribulose-5-phosphate (pentose phosphate pathway) to glucosamine-6-phosphate (GlcN6P) by way of d-fructose-6-phosphate (glycolysis), were upregulated (see Fig. S2C and S3C in the supplemental material). In addition, the *sa2127* (encoding ribose-5-phosphate isomerase A), *fbp*-like (encoding fructose-bisphosphatase), *gnd* (encoding 6-phosphogluconate dehydrogenase), and *pgi* (encoding glucose-6-phosphate isomerase) genes were downregulated in ΔIP1, as shown in Fig. S2C in the supplemental material. Interestingly, in ΔIP2, ΔIP3, and ΔIP4, carrying the *msrR*(E146K) mutation, the levels of transcripts of the *glmS* gene increased. Contrary to expectations, the *gntP* (encoding gluconate permease), *gntK* (encoding glucokinase), *gntR* (encoding the gluconate operon transcriptional repressor), and *fbp* genes were upregulated in ΔIP5 and Mu50 but not in Mu3 or the other ΔIP-derived strains. The profiles in slow VISA strain ΔIP *rpoB*(R512P) were also different from those in the other strains (see Fig. S2C and S3C in the supplemental material).

Comparison of the differences in the transcriptional profiles between strains induced with vancomycin and strains not induced with vancomycin showed that the levels of transcripts of the *gntR* and *gntK* genes increased in ΔIP4 induced with vancomycin but not in ΔIP4 not induced with vancomycin (see Fig. S2B and S3C in the supplemental material). The transcriptional profile in ΔIP4 induced with vancomycin was similar to that in ΔIP5 and Mu50.

## DISCUSSION

The VISA phenotype of Mu50 was successfully reconstituted in VSSA strain N315ΔIP by the sequential introduction of six mutations, i.e., *vraS*(S329L), *msrR*(E146K), *graR*(N197S), *rpoB*(H481Y), *fdh2*(A297V), and *sle1*(Δ67aa). Our study had six major findings. First, we proved the stepwise increase in phenotypic resistance from VSSA strain ΔIP to Mu50-like VISA, by way of hVISA, using an isogenic library. This finding supports the hypothesis that gene mutations led by pressure from antibiotic therapy of MRSA infections constructed a phenotypic VISA strain from a MRSA (VSSA) strain in a hospital. In practical terms, we also revealed that exposure not only to vancomycin but also to imipenem and rifampin selected for hVISA strains carrying *vraS*(S329L) in this study (10).

Second, the results shown in Table 2 indicate that naive *vraS* mutants (ΔIP derivatives) and naive *msrR* mutants (ΔIP1 derivatives) did not convert to phenotypic VISA or hVISA from VSSA. In contrast, mutants with both *vraS*(S329L) and *msrR*(E146K) had a tendency to convert to an hVISA phenotype similar to that of hVISA strain Mu3. When mutants with both *vraS*(S329L) and *msrR*(E146K) were selected by rifampin (Table 5), the level of vancomycin resistance increased, which was indicated by the high frequencies of vancomycin resistance in 11/20 (55%) and 12/20 (60%) isolates of the ΔIP2 and ΔIP3 mutants, respectively. It was suggested that both the *vraS*(S329L) and *msrR*(E146K) mutations are essential for conversion to phenotypic VISA by rifampin. As the *msrR*(E146K) or *vraS*(S329L) mutation was present in 19.4 and 15.8% of clinically isolated hVISA and VISA strains, respectively (11), clinicians should be aware of these findings before prescribing rifampin therapy.

**FIG 5:**
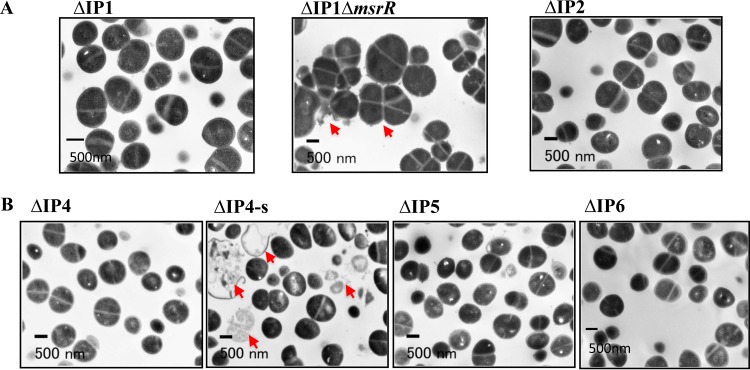
Abnormal morphology of the ΔIP1 Δ*msrR* and ΔIP4-s mutant strains in the absence of vancomycin. Cells were analyzed by transmission electron microscopy. All strains were grown from small inocula under the same standard conditions in BHI medium free of vancomycin. The morphology of the cells in the bacterial cultures was observed by phase-contrast microscopy, and the cells were also analyzed by transmission electron microscopy. In comparison with the morphology of the parental strain, the cultures of ΔIP1, ΔIP2, ΔIP4, and ΔIP5 were composed of regularly shaped and well-separated cocci, and cultures of the ΔIP1 Δ*msrR* and ΔIP4-s strains grew as multicellular aggregates. Electron microscopic sections of ΔIP1 Δ*msrR* and ΔIP4-s show clusters of unseparated cells with abnormally thick cell walls, and the cells are also surrounded by a large amount of amorphous extracellular material (red arrows). However, cells of the *msrR*(E146K) mutant strain are normally shaped. Δ*sle1* mutant strain ΔIP4-s failed to separate and had a much thicker cell wall than Mu50, although the cell walls of ΔIP5 and ΔIP6 showed a normal separation that was the same as that of Mu50.

Third, *msrR*(E146K) is responsible for conversion to the Mu3-type hVISA phenotype. *msrR* regulates genes encoding cell wall synthesis and antibiotic resistance: *hmrA*, *sarH*, *sigB*, etc. However, the data indicate that *msrR*(E146K) downregulated several genes related to cell wall and WTA synthesis (see Fig. S2A and S3A in the supplemental material). *msrR*, which is a member of the LytR-CspA-Psr (LCP) family of membrane proteins, contributes to the development of high-level resistance to β-lactam antibiotics (37) and cell surface characteristics and virulence in S. aureus (38, 39). In this study, we also demonstrated that the *msrR*(E146K) mutation influences resistance to β-lactams and glycopeptides (Table 2), as reported previously (7). The Δ*msrR* mutation led to reduced resistance to oxacillin (Table 2), and the cells failed to separate fully and showed aberrant septal placement (Fig. 5). Chan et al. reported that a Δ*lcp* mutant showed a defect in tethering WTA to the cell wall; cleaved WTA synthesis intermediates, releasing ribitol phosphate into the medium; and recycled bactoprenol for peptidoglycan synthesis (40). It has been reported that *lcp*, including *msrR* of S. aureus, encodes promiscuous enzymes that attach secondary cell wall polymers with discrete linkage units to peptidoglycan (40). Thus, it was suggested that the *msrR*(E146K) mutation promotes the tethering of WTA and the capsule to the cell wall, which then leads to decreased autolytic activity and resistance to vancomycin.

Fourth, the *rpoB*(H481Y) mutation was related not only to an increase in vancomycin resistance but also to a low growth rate and decreased autolysin activity (Fig. 4 and Table 4). The *rpoB*(H481Y) mutation was also previously related to an increase in resistance to vancomycin (10, 17, 21). For the subpopulation for which the vancomycin MIC was 4 μg/ml, for example, for the ΔIP1-r strain, the number of CFU was 5 × 10^2^, which was similar to the value for hVISA strain Mu3. The *rpoB*(H481Y) mutation has been found in Mu50 and 70% of VISA strains throughout the world (41) and is related to the increase in the rates of resistance to vancomycin and daptomycin (10). Recently, we have also reported that the RNA polymerase mutation *rpoB*(R512P) acts on reversible resistance to vancomycin (19) and seems to have a regulatory effect in triggering the entire scheme of peptidoglycan synthesis. We have reported that the *rpoB* and *rpoC* mutations are regulatory mutations (17, 19, 42, 43). It was supposed that the *rpoB* mutation acts as a regulatory mutation in the acquisition of the VISA phenotype.

The *rpoB*(H481Y) mutants ΔIP4, ΔIP5, and Mu50 showed significant downregulation of the *pyr* genes. The products of the *pyr* operon are involved in the *de novo* synthesis of pyrimidine nucleotides from bicarbonate and from intermediates of central carbon metabolism or via salvage of preformed pyrimidine bases and nucleotides present in the medium. The *pyr* operon is regulated by transcription attenuation in response to exogenous uracil, which was determined using transcription studies and determination of the intracellular pyrimidine triphosphate nucleoside, UDP, and UMP/phosphoribosyl pyrophosphate (PRPP) pool size (44). In Bacillus subtilis, PyrR controls the expression of the *pyr* operon by binding to specific sequences of *pyr* mRNA, thereby leading to the attenuation of transcription (45, 46) in response to exogenous uracil and to intracellular PRPP pools (described below).

Moreover, it was found for the first time that vancomycin induced an increase in cell wall thickness in ΔIP4. The thickening of the cell wall of ΔIP4 was increased by induction with vancomycin at 10 μg/ml, although the cell walls of ΔIP5 and Mu50 were thickened constitutively.

**FIG 6:**
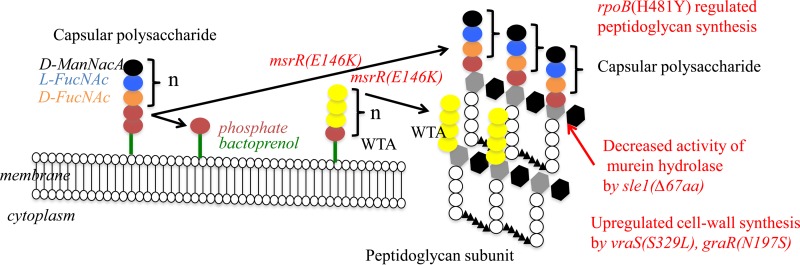
Possible effect of mutations in six genes on Mu50-type vancomycin resistance. (i) In ΔIP1, upregulated cell wall synthesis results in an increase in cell wall thickness. (ii) In ΔIP2, autolytic activity is decreased, and because of the deposition of LysM-type murein hydrolase, the cell division type was restricted and tethering of WTA and capsular polysaccharide to the cells was increased. (iii) In ΔIP3, upregulated cell wall synthesis results in an increase in cell wall thickness and conversion from the hVISA to VISA phenotype. (iv) In ΔIP4, a regulatory mutation resulted in slow growth and an increase in cell wall thickness. (v) In ΔIP5, resistance to vancomycin occurred with the presence of other mutated genes. The function is unknown. It seems that the *fdh2*(A297V) mutation regulates the moderate activity of the Δ*sle1* mutant. (vi) In ΔIP6, the activity of LysM-type murein hydrolase (autolysis) was decreased, resulting in an increase in cell wall thickness and resistance to vancomycin.

Fifth, we verified the effects of the *msrR*(E146K) and *sle1*(Δ67aa) mutations. The deposition of LysM murein hydrolases of *sle1* in the envelope of Δ*lcp* mutant staphylococci was detected (Fig. 6). The decoration of staphylococcal peptidoglycan with WTA restricts the binding of secreted murein hydrolases to the cell wall and limits the autolytic activity of these enzymes on the cross-wall compartment of dividing staphylococci. These data suggest that the WTA deposition defect of the Δ*lcp* mutant with the Δ*msrR* mutation causes the unrestricted deposition of murein hydrolases in the bacterial envelope, a phenotype that likely contributes to the decreased viability of the Δ*msrR* daughter cells generated during cell division.

Finally, several other genes involved in cell wall synthesis and modification were found to respond to vancomycin. GlmS activity has a critical role in the initiation of PG synthesis, which increased in the ΔIP1, ΔIP2, ΔIP3, and ΔIP4 strains without vancomycin. It has recently been reported that the *glmS* riboswitch is unique among riboswitch families, as it represents a metabolite-dependent ribozyme that undergoes self-cleavage upon recognition of glucosamine-6-phosphate (GlcN6P) (47, 48). In contrast to the findings for ΔIP4 with induction by vancomycin, the ΔIP5 and Mu50 VISA strains showed the upregulation of the *gntR*, *gntP*, *gntK*, and *ndk* genes instead of the *glmS* gene and the downregulation of the *cmk* and *pyr* genes (described above), which are associated with the thickness of the cell wall and the size of the UTP and UMP pool (49).

The gluconate (*gnt*) operon of Bacillus subtilis includes the *gntR*, *gntK*, *gntP*, and *gntZ* genes, encoding the transcriptional repressor of the operon, gluconate kinase, the gluconate permease, and an unidentified open reading frame, respectively (50). GntR is classified in the GntR/DeoR family, which includes the Gnt-like protein LacR, and LacR is involved in the repression of the genes for lactose and fructose in Lactococcus lactis (51). Also, the GntR family repressor YtrA responds to the cell wall antibiotics ramoplanin and moenomycin, which are involved in binding to the substrate lipid II and the transglycosylase enzyme, respectively, in Bacillus subtilis (52). In the case of S. aureus, it has been reported that the GntR-like protein NorG, which positively affects the transcription of global regulators, has been shown to affect S. aureus genes involved in resistance to quinolones and β-lactams, such as genes encoding the NorB and AbcA transporters (53). It is possible that the Gnt operon might be related to cell wall metabolism in ΔIP5 and Mu50; however, the role of the Gnt operon is unknown.

The *ndk* gene encodes nucleoside diphosphate kinase, catalyzes the synthesis of the nucleoside triphosphates UTP and CTP, and then maintains intracellular triphosphate pools in E. coli (54). The loss of function of Ndk in E. coli results in an increased rate of appearance of rifampin-resistant strains and an imbalance in deoxynucleoside triphosphate pool levels, and Ndk prevents the accumulation of dUTP in the cell (55). It seems that the size of the UTP pool increased.

In fact, we also reported that the *cmk* mutation leads to a thickening of cell wall peptidoglycan layers, increasing vancomycin resistance (17). When a metabolomics analysis between hVISA Mu3 and slow VISA Mu3-6R was performed, the amounts of UDP, UTP, and UDP-*N*-acetylglucosamine (UDP-GlcNAc) produced from GlcNAc-1P and UTP (56) were increased and the amount of GlcNAc-1P was decreased in Mu3-6R (Y. Katayama, unpublished data). Therefore, we suppose that the increase in the amount of UTP in the cell promotes cell wall synthesis.

In conclusion, this study provides new and important information on the functional significance of the mechanism responsible for increased resistance to vancomycin in strains of the Mu50 type. All six mutated genes required for acquisition of the VISA phenotype were directly or indirectly involved in the regulation of cell physiology. VISA seemed to be achieved through multiple genetic events accompanying drastic changes in cell physiology.

## Supplementary Material

Supplemental material
