# Supplementary material for: Complete Reconstitution of the Vancomycin-Intermediate Staphylococcus aureus Phenotype of Strain Mu50 in Vancomycin-Susceptible S. aureus
Source: Antimicrob Agents Chemother. 2016 May 23;60(6):3730–42. doi: 10.1128/AAC.00420-16 (PMC4879404; doi:10.1128/AAC.00420-16)
Supplement: Supplemental material [file supp_60_6_3730__index.html]

Complete Reconstitution of the Vancomycin-Intermediate Staphylococcus aureus Phenotype of Strain Mu50 in Vancomycin-Susceptible S. aureus — Supplemental material 

# Complete Reconstitution of the Vancomycin-Intermediate Staphylococcus aureus Phenotype of Strain Mu50 in Vancomycin-Susceptible S. aureus

## Supplemental material

- Supplemental file 1 -

  Fig. S1-S3

  PDF, 1.7M
