# Supplementary material for: Complete Reconstitution of the Vancomycin-Intermediate Staphylococcus aureus Phenotype of Strain Mu50 in Vancomycin-Susceptible S. aureus
Source: Antimicrob Agents Chemother. 2016 May 23;60(6):3730–42. doi: 10.1128/AAC.00420-16 (PMC4879404; doi:10.1128/AAC.00420-16)
Supplement: Supplemental material [file AAC.00420-16_zac006165257so1.pdf]

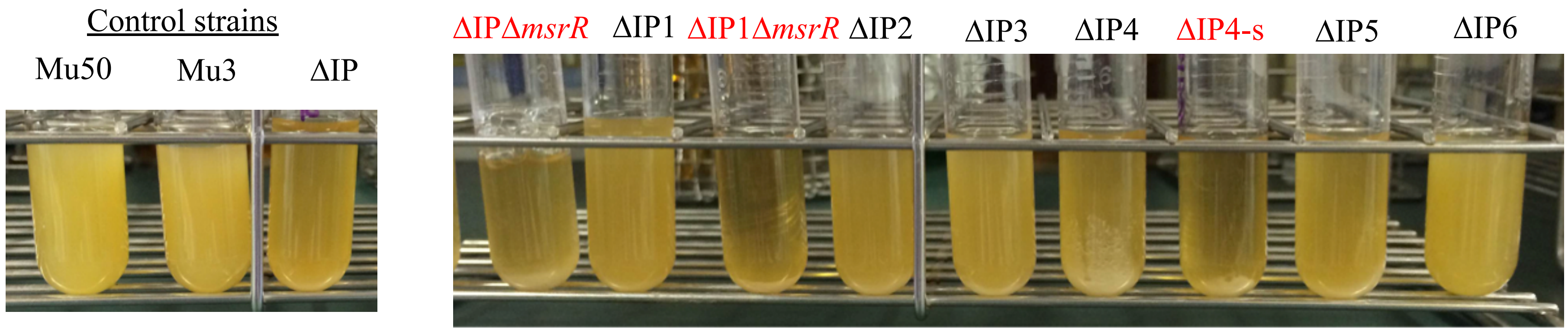

Figure S1. Abnormal morphology of ΔIP1Δ*msrR* and ΔIP4-s(=ΔIP4Δ*sle1*) mutant strains in the absence of vancomycin. Cell aggregation phenotype in BHI broth. All strains were grown from small inocula in the same standard conditions in BHI free of vancomycin. In comparison with the parental strain ΔIP1, ΔIP2, ΔIP4 and ΔIP5, the cultures of which were composed of regularly shaped and well separated cocci, cultures of the ΔIP1Δ*msrR* and ΔIP4-s grew as multicellular aggregates.

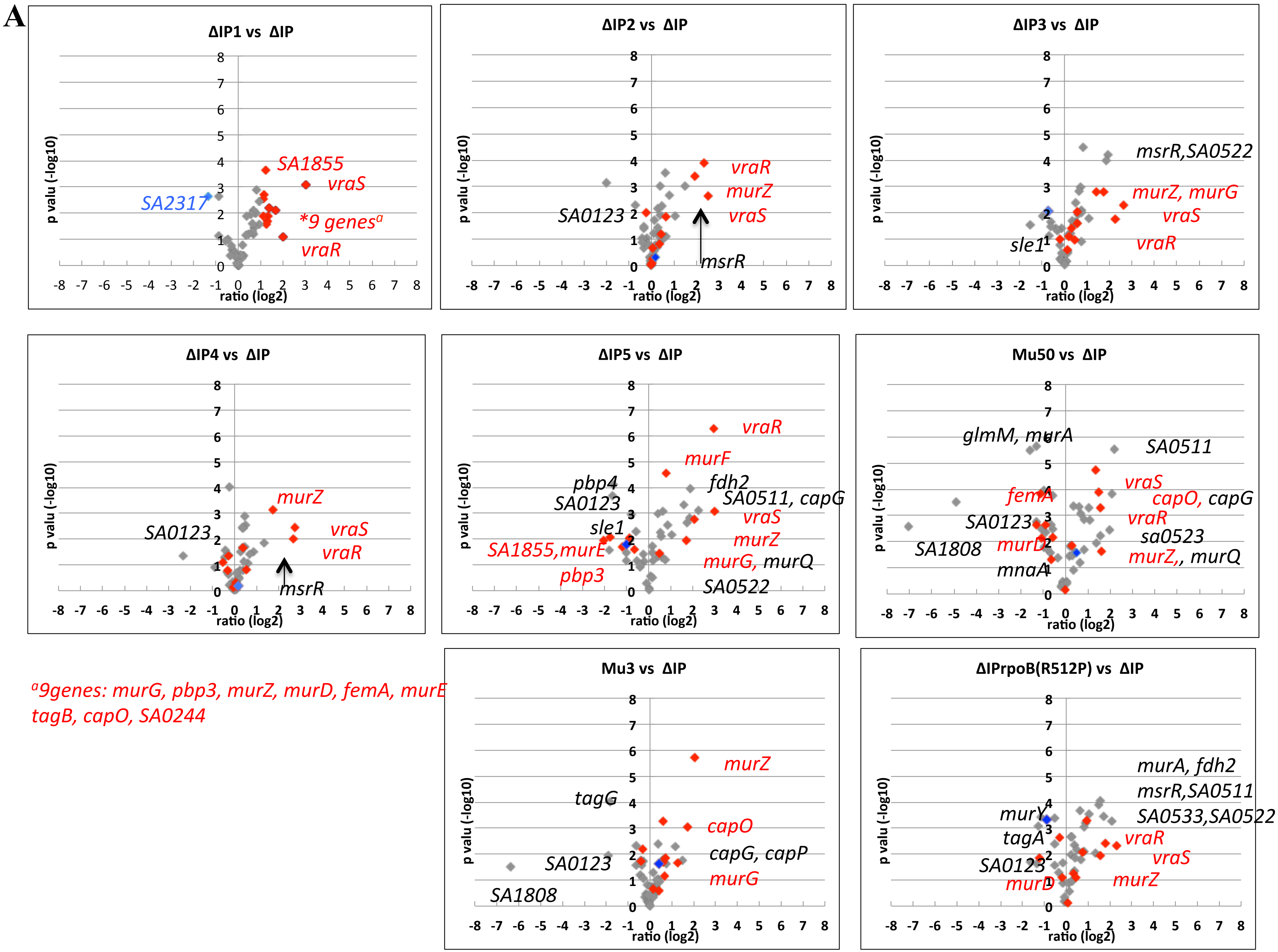
A
ΔIP1 vs ΔIP
p valu (-log10)
ratio (log2)
SA1855
vraS
SA2317
*9 genes[a]
vraR
ΔIP2 vs ΔIP
vraR
murZ
SA0123
vraS
msrR
ΔIP3 vs ΔIP
msrR,SA0522
murZ, murG
vraS
sle1
vraR
ΔIP4 vs ΔIP
murZ
SA0123
vraS
vraR
msrR
ΔIP5 vs ΔIP
vraR
murF
pbp4
fdh2
SA0123
SA0511, capG
sle1
vraS
murZ
SA1855,murE
murG, murQ
pbp3
SA0522
Mu50 vs ΔIP
glmM, murA
SA0511
vraS
femA
capO, capG
SA0123
vraR
sa0523
SA1808
murD
murZ,, murQ
mnaA
[a]9genes: murG, pbp3, murZ, murD, femA, murE
tagB, capO, SA0244
Mu3 vs ΔIP
murZ
tagG
capO
capG, capP
SA0123
murG
SA1808
ΔIPrpoB(R512P) vs ΔIP
murA, fdh2
msrR,SA0511
SA0533,SA0522
murY
tagA
vraR
vraS
SA0123
murD
murZ

B

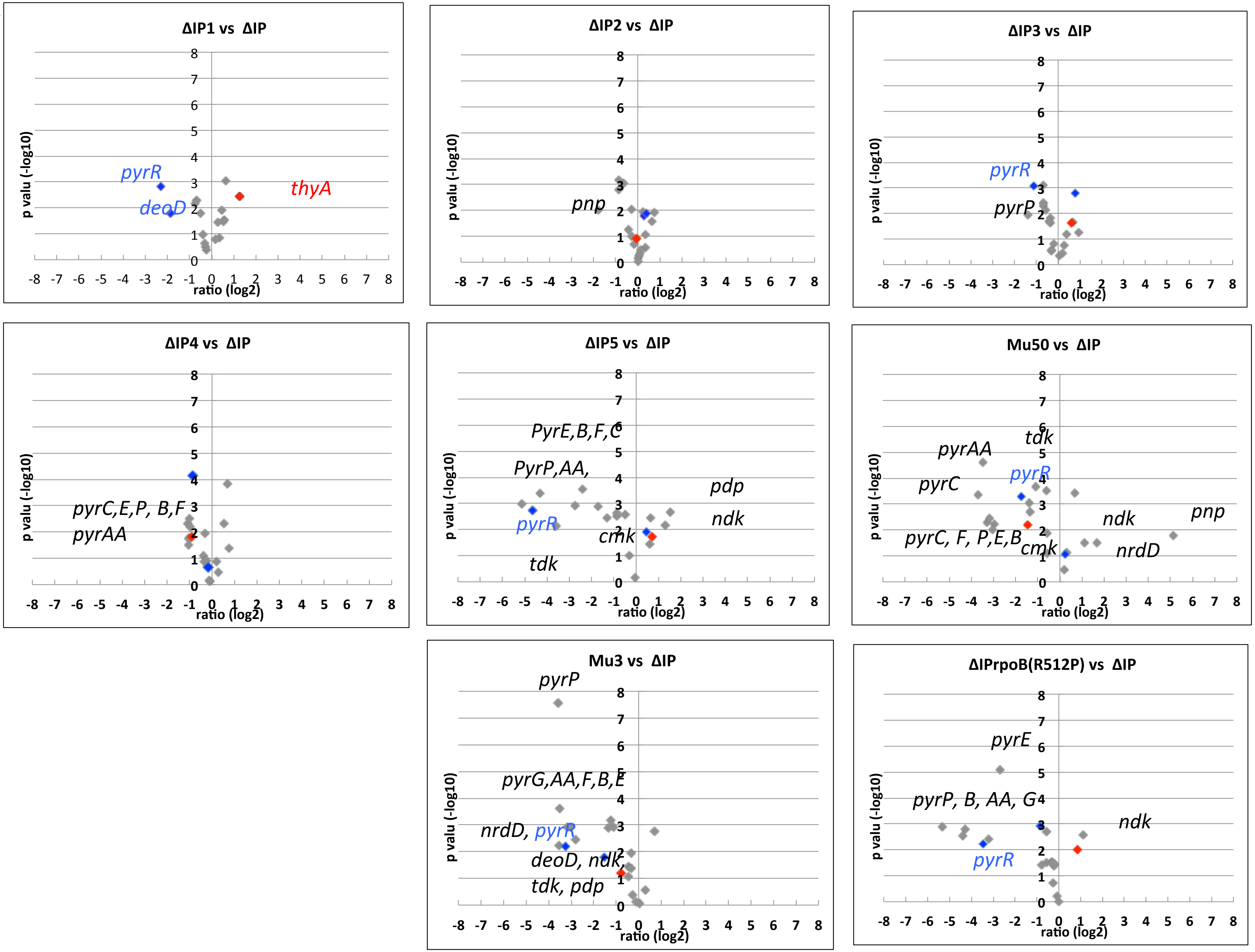
ΔIP1 vs ΔIP
pyrR
deoD
thyA
p valu (-log10)
ratio (log2)
ΔIP2 vs ΔIP
pnp
p valu (-log10)
ratio (log2)
ΔIP3 vs ΔIP
pyrR
pyrP
p valu (-log10)
ratio (log2)
ΔIP4 vs ΔIP
pyrC,E,P, B,F
pyrAA
p valu (-log10)
ratio (log2)
ΔIP5 vs ΔIP
PyrE,B,F,C
PyrP,AA,
pyrR
cmk
tdk
pdp
ndk
p valu (-log10)
ratio (log2)
Mu50 vs ΔIP
pyrAA
tdk
pyrC
pyrR
ndk
pnp
pyrC, F, P,E,B
cmk
nrdD
p valu (-log10)
ratio (log2)
Mu3 vs ΔIP
pyrP
pyrG,AA,F,B,E
nrdD, pyrR
deoD, ndk
tdk, pdp
p valu (-log10)
ratio (log2)
ΔIPrpoB(R512P) vs ΔIP
pyrE
pyrP, B, AA, G
ndk
pyrR
p valu (-log10)
ratio (log2)

C

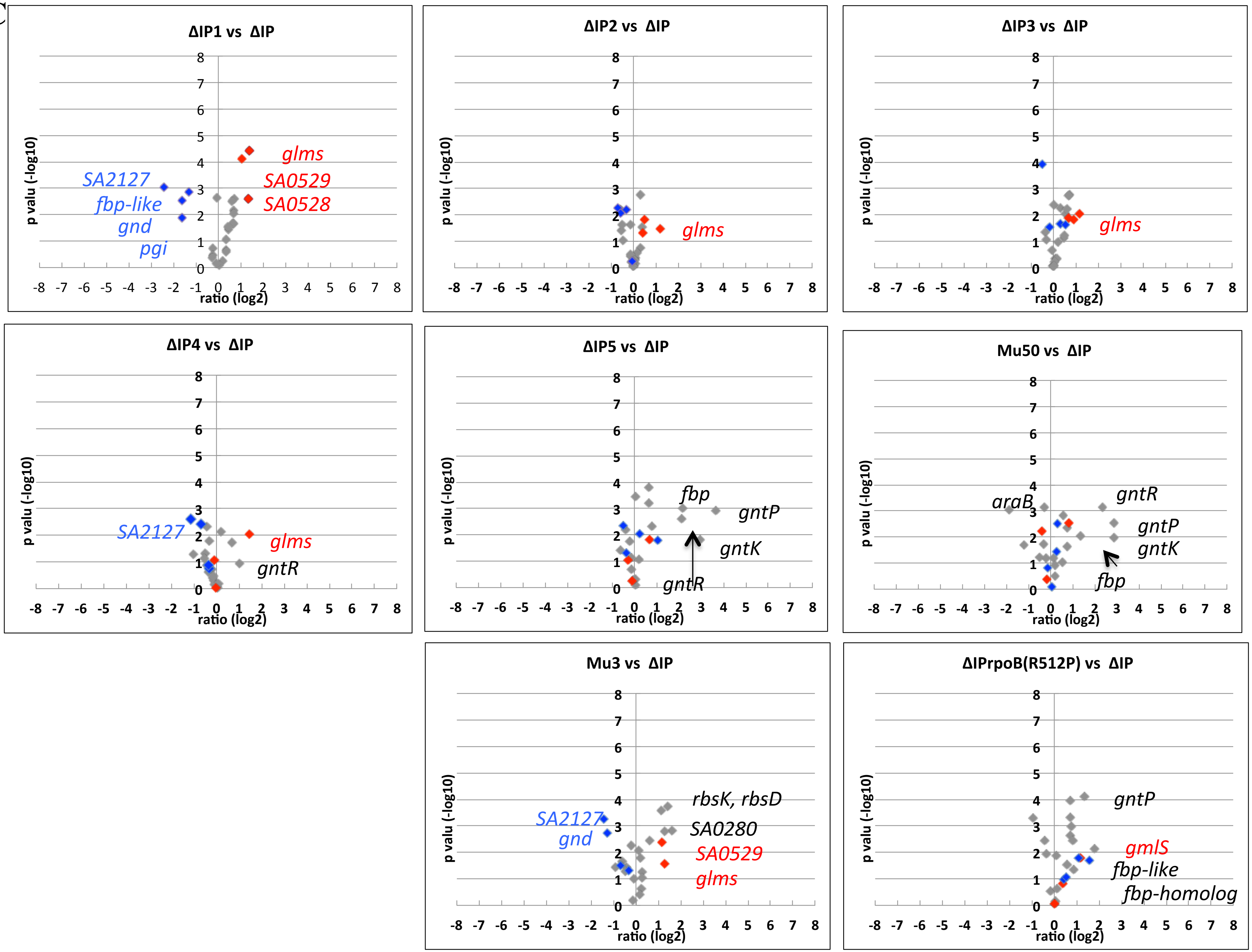
ΔIP1 vs ΔIP
p valu (-log10)
ratio (log2)
glms
SA0529
SA0528
SA2127
fbp-like
gnd
pgi
ΔIP2 vs ΔIP
p valu (-log10)
ratio (log2)
glms
ΔIP3 vs ΔIP
p valu (-log10)
ratio (log2)
glms
ΔIP4 vs ΔIP
p valu (-log10)
ratio (log2)
SA2127
glms
gntR
ΔIP5 vs ΔIP
p valu (-log10)
ratio (log2)
fbp
gntP
gntK
gntR
Mu50 vs ΔIP
p valu (-log10)
ratio (log2)
araB
gntR
gntP
gntK
fbp
Mu3 vs ΔIP
p valu (-log10)
ratio (log2)
rbsK, rbsD
SA0280
SA0529
glms
SA2127
gnd
ΔIPrpoB(R512P) vs ΔIP
p valu (-log10)
ratio (log2)
gntP
gmlS
fbp-like
fbp-homolog

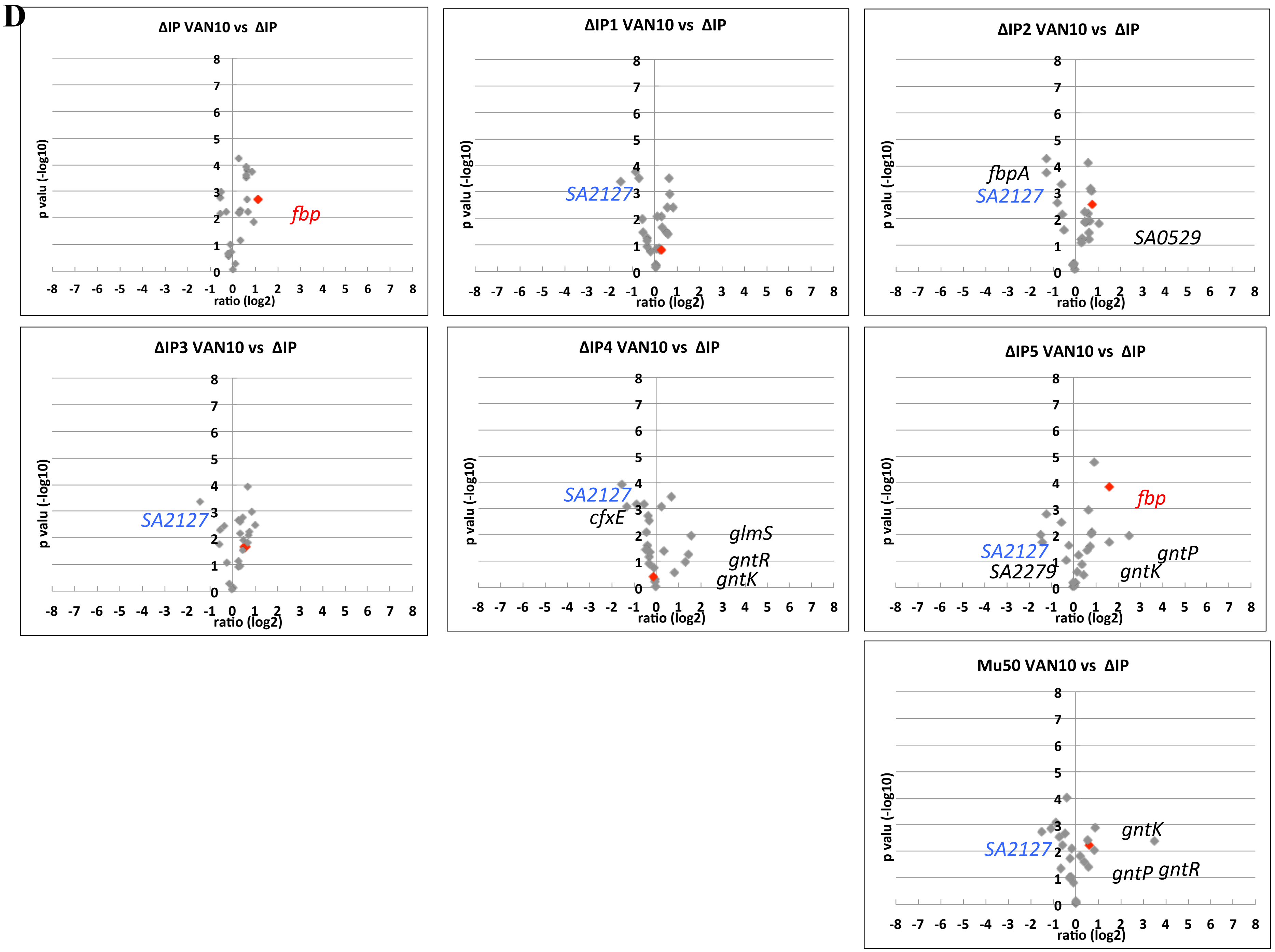
D
ΔIP VAN10 vs ΔIP
fbp
ΔIP1 VAN10 vs ΔIP
SA2127
ΔIP2 VAN10 vs ΔIP
fbpA
SA2127
SA0529
ΔIP3 VAN10 vs ΔIP
SA2127
ΔIP4 VAN10 vs ΔIP
SA2127
cfxE
glmS
gntR
gntK
ΔIP5 VAN10 vs ΔIP
fbp
SA2127
SA2279
gntP
gntK
Mu50 VAN10 vs ΔIP
gntK
SA2127
gntP gntR
p valu (-log10)
ratio (log2)

Figure S2. Volcano plot analysis of the transcriptional profiles, of which (A) major 51 genes related to the peptidoglycan, wall-teichoic acid and capsular polysaccharide synthesis shown in figure 8A, and *vraSR, msrR, graR, fdh2* and *sle1* genes, (B) 19 genes in pyrimidine metabolic pathway, (C and D) 27 genes related to the metabolic pathway from pentose phosphate pathway to cell-wall synthesis by way of glycolysis. (A and B) hVISA strain Mu3, VISA strain Mu50 and slow-VISA ΔIP*rpoB*(R512P) were used as controls. The $-\log_{10}$ (*p*-value for a *t*-test) values are plotted against the log rations ($\log_2$ fold change). The blue or red plots indicated that the genes down- or up-regulated by greater than 2-fold in ΔIP1 /ΔIP (A, B and C) and ΔIP induced by VAN10/ΔIP without VAN 10 (D). The grey plots showed the genes having *p*-value $\geq$ 0.05 with *t*-test. . Each strain was cultured with 4 mL of BHI (A, B and C) in the absent of antibiotics.

(A) The level of transcripts of 17 genes (indicated by red) were increased in ΔIP1 carrying *vraS* mutation. The transcripts of *msrR* (E146K) and *fdh* (A297V) genes were increased in ΔIP2, ΔIP3, ΔIP4 and ΔIP5. (B) Effect of *fdh2(A297V)* gene mutation in ΔIP5 on transcriptional profiles. The transcriptional profiles of ΔIP5 were compared to the other ΔIP derived-mutant strain and clinical VISA strain Mu50. Note that *gntR, gntP* and *gntK* (encoding D-gluconate permease and phosphorylation from D-gluconate to D-gluconate-6P), and *fbp* (encoding fructose-bisphophatase) were up-regulated by greater than 2-fold in the both of ΔIP5 and Mu50, compared to those in other ΔIP derived-mutant strains. In contract, ΔIP1, ΔIP2, ΔIP3 and ΔIP4 showed that the level of transcription in *glms* gene increased, as compared to those in other strains. (C) The comparison of transcriptional profile of ΔIP4 between with induction by VAN10 and drug-free. The cell-wall thickening of ΔIP4 was increased by induction of VAN 10 μg/mL same as that of Mu50, but those of ΔIP5 were constitutively thickened cell-wall. Note that the *gntR, gntP* and *gntK* were up-regulated, and *sa2107* was down-regulated in the ΔIP4 with VAN 10 μg/mL, but not shown in those without VAN10 μg/mL. (D)

A

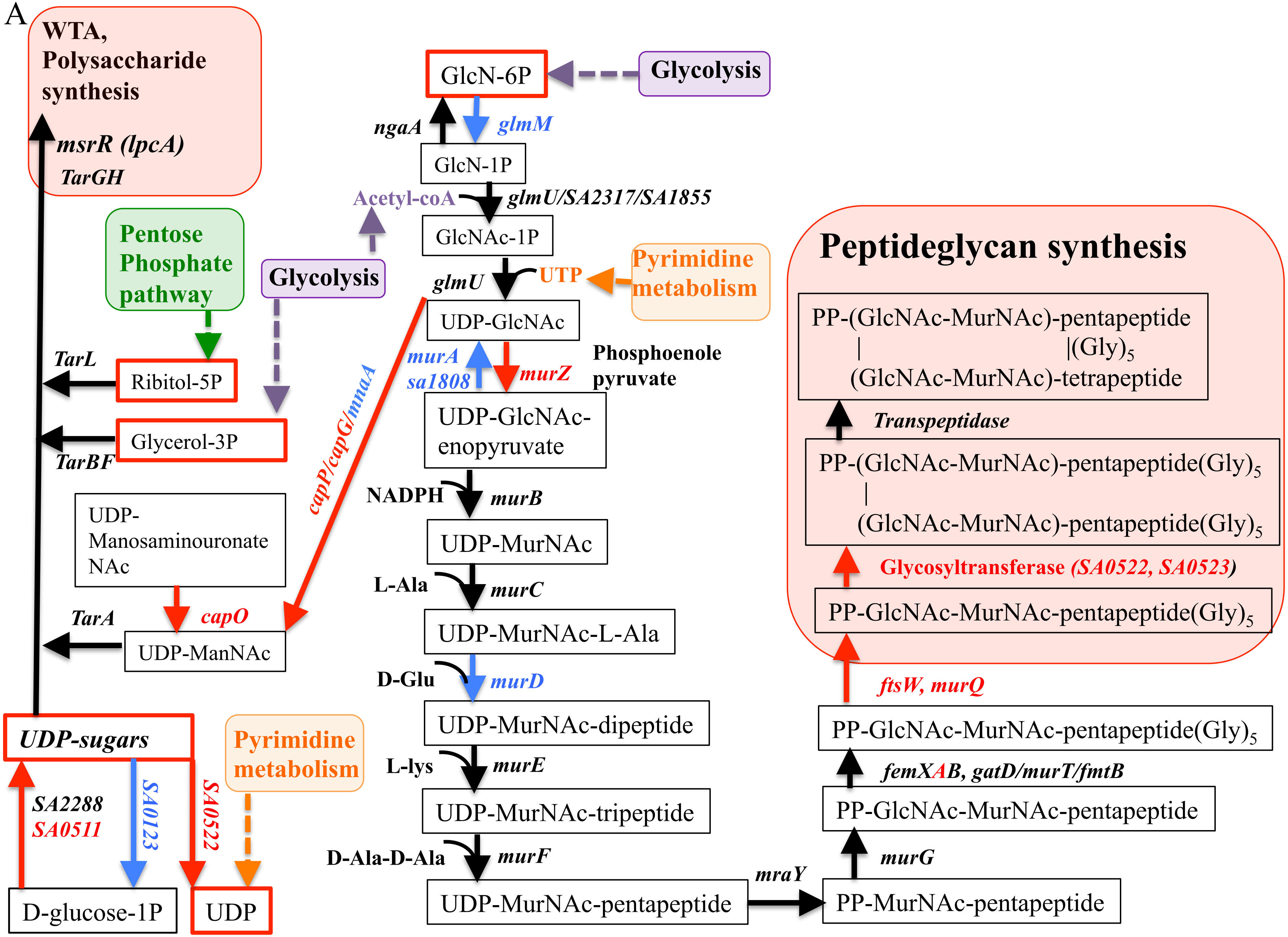
WTA,
Polysaccharide
synthesis
msrR (lpcA)
TarGH
GlcN-6P
Glycolysis
ngaA
glmM
GlcN-1P
Acetyl-coA
glmU/SA2317/SA1855
GlcNAc-1P
Pentose
Phosphate
pathway
Glycolysis
glmU
UTP
Pyrimidine
metabolism
UDP-GlcNAc
Peptideglycan synthesis
PP-(GlcNAc-MurNAc)-pentapeptide
|(Gly)5
(GlcNAc-MurNAc)-tetrapeptide
TarL
Ribitol-5P
murA
sa1808
murZ
Phosphoenole
pyruvate
Glycerol-3P
TarBF
capP/capG/mnaA
UDP-GlcNAc-
enopyruvate
Transpeptidase
PP-(GlcNAc-MurNAc)-pentapeptide(Gly)5
(GlcNAc-MurNAc)-pentapeptide(Gly)5
NADPH
murB
UDP-
Manosaminouronate
NAc
UDP-MurNAc
Glycosyltransferase (SA0522, SA0523)
L-Ala
murC
PP-GlcNAc-MurNAc-pentapeptide(Gly)5
TarA
capO
UDP-MurNAc-L-Ala
UDP-ManNAc
D-Glu
murD
ftsW, murQ
UDP-MurNAc-dipeptide
PP-GlcNAc-MurNAc-pentapeptide(Gly)5
UDP-sugars
Pyrimidine
metabolism
L-lys
murE
femXAB, gatD/murT/fmtB
SA2288
SA0511
SA0123
SA0522
UDP-MurNAc-tripeptide
PP-GlcNAc-MurNAc-pentapeptide
D-Ala-D-Ala
murF
murG
mraY
D-glucose-1P
UDP
UDP-MurNAc-pentapeptide
PP-MurNAc-pentapeptide

B

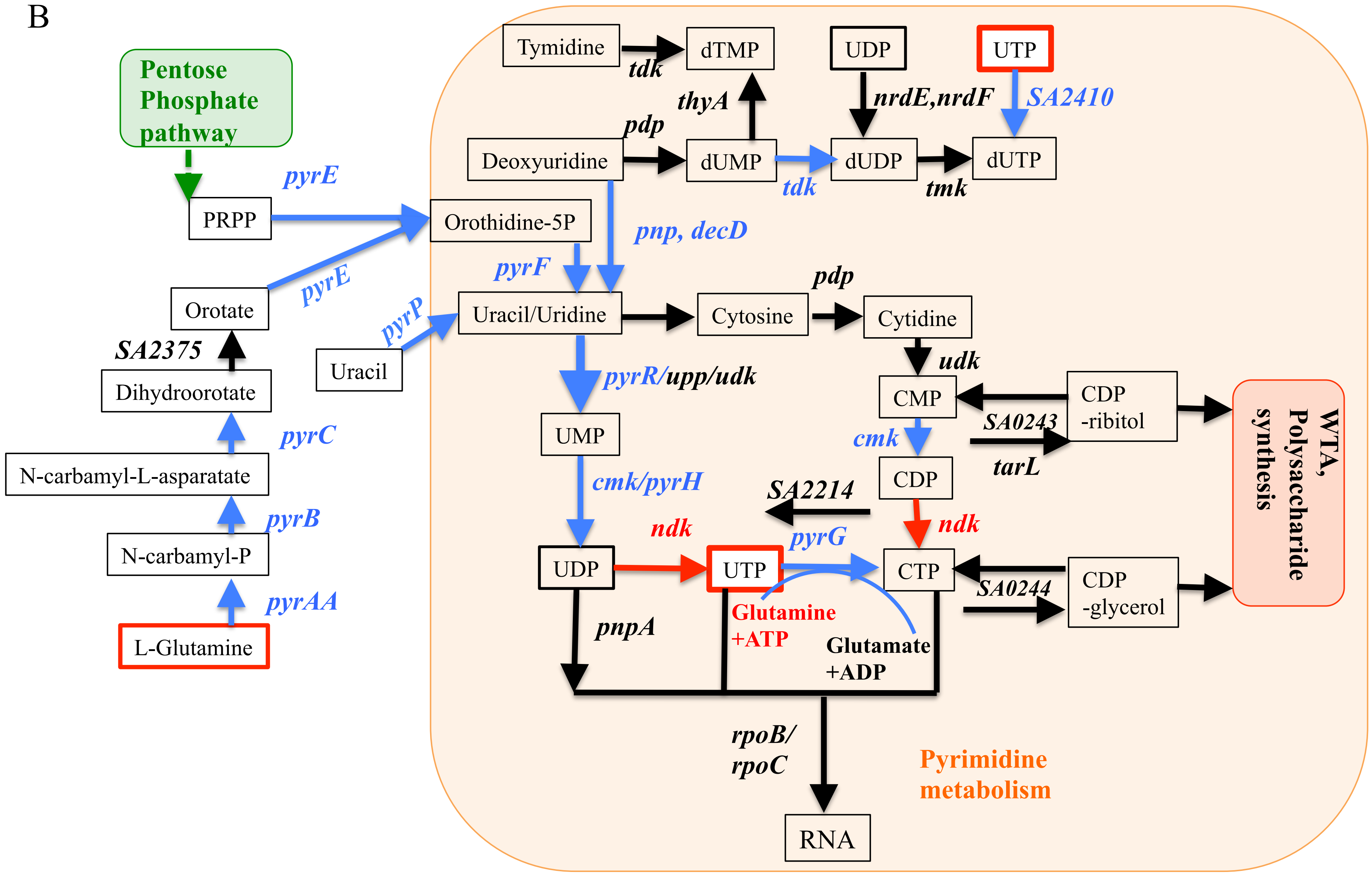

Pentose Phosphate pathway
PRPP
pyrE
Orothidine-5P
Orotate
pyrE
SA2375
Dihydroorotate
pyrC
N-carbamyl-L-asparatate
pyrB
N-carbamyl-P
pyrAA
L-Glutamine
Uracil
pyrP
Tymidine
tdk
dTMP
thyA
pdp
Deoxyuridine
dUMP
tdk
UDP
nrdE,nrdF
dUDP
UTP
SA2410
dUTP
tmk
pnp, decD
pyrF
Uracil/Uridine
Cytosine
pdp
Cytidine
udk
pyrR/upp/udk
UMP
CMP
CDP -ribitol
SA0243
tarL
cmk
CDP
cmk/pyrH
SA2214
ndk
pyrG
UDP
ndk
UTP
CTP
SA0244
CDP -glycerol
WTA, Polysaccharide synthesis
pnpA
Glutamine +ATP
Glutamate +ADP
rpoB/ rpoC
RNA
Pyrimidine metabolism

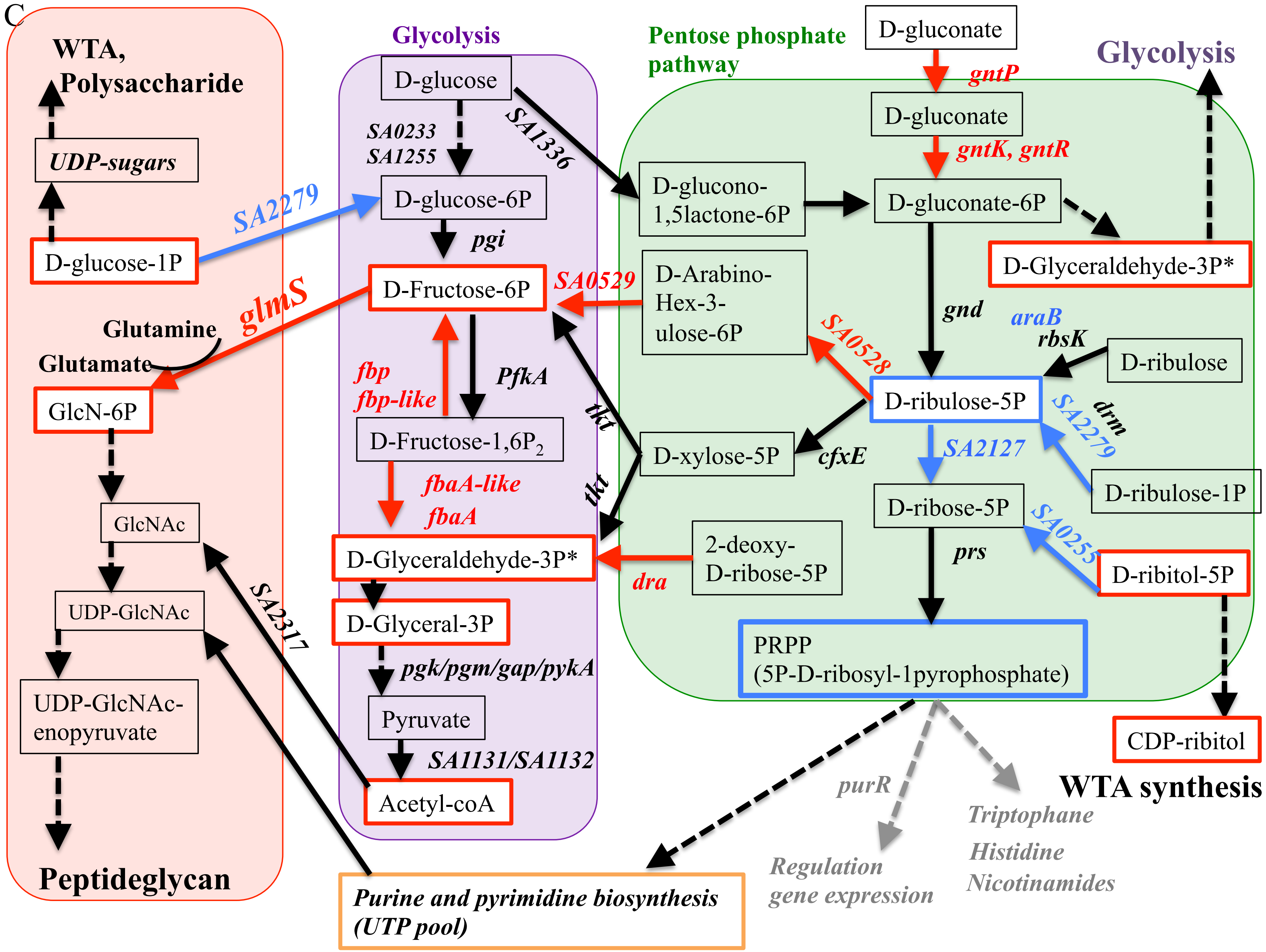
C
WTA, Polysaccharide
UDP-sugars
D-glucose-1P
SA2279
glmS
Glutamine
Glutamate
GlcN-6P
GlcNAc
UDP-GlcNAc
UDP-GlcNAc-enopyruvate
Peptideglycan
SA2317
Glycolysis
D-glucose
SA0233
SA1255
SA1336
D-glucose-6P
pgi
D-Fructose-6P
fbp
fbp-like
PfkA
D-Fructose-1,6P2
fbaA-like
fbaA
D-Glyceraldehyde-3P*
D-Glyceral-3P
pgk/pgm/gap/pykA
Pyruvate
SA1131/SA1132
Acetyl-coA
Pentose phosphate pathway
D-gluconate
gntP
D-gluconate
gntK, gntR
D-glucono-1,5lactone-6P
D-gluconate-6P
Glycolysis
D-Glyceraldehyde-3P*
SA0529
D-Arabino-Hex-3-ulose-6P
SA0528
gnd
araB
rbsK
D-ribulose
D-ribulose-5P
drm
SA2279
tkt
tkt
D-xylose-5P
cfxE
SA2127
D-ribose-5P
D-ribulose-1P
SA0255
2-deoxy-D-ribose-5P
dra
prs
D-ribitol-5P
PRPP (5P-D-ribosyl-1pyrophosphate)
CDP-ribitol
WTA synthesis
purR
Triptophane
Histidine
Nicotinamides
Regulation gene expression
Purine and pyrimidine biosynthesis (UTP pool)

Figure S3. (A) Metabolic pathway of cell-wall biosynthesis. Peptidoglycan and wall-teichoic acids (WTAs) biosynthesis are two main processes of the cell-wall biosynthesis pathway. (B) The pathway of pyrimidine metabolism. **(C)**The pathway of Pentose phosphate metabolism, central carbon pathways and peptidoglycan synthesis in *S. aureus*. The arrows correspond to potential active enzymatic reactions catalysed by the corresponding genes products encoded by the *S. aureus* genome data of KEGG (http://www.genome.jp/kegg-bin/show_pathway?sau00030+SA2304). The area of glycolysis biosynthesis, peptidoglycan and pentose phosphate pathway were indicated by purple, red and green, respectively. SA2137 is classified into similar to N-acetyltransferase.
